# Benthic Dissolved Silicon and Iron Cycling at Glaciated Patagonian Fjord Heads

**DOI:** 10.1029/2022GB007493

**Published:** 2022-11-25

**Authors:** Hong Chin Ng, Jon R. Hawkings, Sebastien Bertrand, Brent A. Summers, Matthias Sieber, Tim M. Conway, Felipe S. Freitas, James P. J. Ward, Helena V. Pryer, Jemma L. Wadham, Sandra Arndt, Katharine R. Hendry

**Affiliations:** ^1^ School of Earth Sciences University of Bristol Bristol UK; ^2^ Ifremer Université Bretagne Occidentale CNRS Geo‐Ocean Plouzané France; ^3^ Department of Earth and Environmental Science University of Pennsylvania Philadelphia PA USA; ^4^ Renard Centre of Marine Geology Ghent University Gent Belgium; ^5^ College of Marine Science University of South Florida St Petersburg FL USA; ^6^ BGeosys Department of Geosciences Université libre de Bruxelles Brussels Belgium; ^7^ Bristol Glaciology Centre School of Geographical Sciences University of Bristol Bristol UK; ^8^ Department of Earth Sciences University of Cambridge Cambridge UK; ^9^ Department of Geosciences Centre for Arctic Gas Hydrate, Environment and Climate (CAGE) UiT The Arctic University of Norway Tromsø Norway; ^10^ Polar Oceans Team British Antarctic Survey Cambridge UK

**Keywords:** fjord biogeochemistry, sediment nutrient cycling, silicon and iron, stable isotopes, early diagenesis, reaction transport modeling

## Abstract

Glacier meltwater supplies silicon (Si) and iron (Fe) sourced from weathered bedrock to downstream ecosystems. However, the extent to which these nutrients reach the ocean is regulated by the nature of the benthic cycling of dissolved Si and Fe within fjord systems, given the rapid deposition of reactive particulate fractions at fjord heads. Here, we examine the benthic cycling of the two nutrients at four Patagonian fjord heads through geochemical analyses of sediment pore waters, including Si and Fe isotopes (δ^30^Si and δ^56^Fe), and reaction‐transport modeling for Si. A high diffusive flux of dissolved Fe from the fjord sediments (up to 0.02 mmol m^−2^ day^−1^) compared to open ocean sediments (typically <0.001 mmol m^−2^ day^−1^) is supported by both reductive and non‐reductive dissolution of glacially‐sourced reactive Fe phases, as reflected by the range of pore water δ^56^Fe (−2.7 to +0.8‰). In contrast, the diffusive flux of dissolved Si from the fjord sediments (0.02–0.05 mmol m^−2^ day^−1^) is relatively low (typical ocean values are >0.1 mmol m^−2^ day^−1^). High pore water δ^30^Si (up to +3.3‰) observed near the Fe(II)‐Fe(III) redox boundary is likely associated with the removal of dissolved Si by Fe(III) mineral phases, which, together with high sedimentation rates, contribute to the low diffusive flux of Si at the sampled sites. Our results suggest that early diagenesis promotes the release of dissolved Fe, yet suppresses the release of dissolved Si at glaciated fjord heads, which has significant implications for understanding the downstream transport of these nutrients along fjord systems.

## Graphical abstract

Schematic shows the coupling of benthic Fe and Si cycling, and the inferred spatial difference in the supply of easily reducible Fe (oxy‐)hydroxides and the intensity of pore water advection between the fjord sites. Adsorption of Si onto Fe (oxy‐)hydroxides and co‐precipitation of amorphous Fe‐Si phases occur under relatively oxic conditions in the upper sediments, while Si is released back to solution following the reductive dissolution of Fe in the ferruginous zone. Circles with brighter colors and solid outlines represent solid‐bound Si and Fe(III) phases, while circles with lighter colors and dashed outlines represent dissolved Si and Fe^2+^ phases.

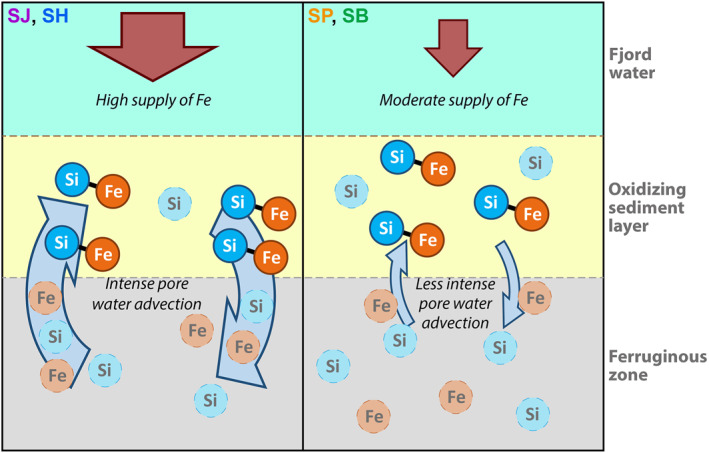

**Key Points**

- Pore water isotope analyses and diagenetic modeling reveal a critical link between benthic silicon and iron cycles at fjord heads
- Benthic fluxes of iron at fjord heads are higher than in the open ocean; the opposite trend is observed for benthic fluxes of silicon
- High levels of reactive iron in fjord sediments restrict the solubility of nutrient silicon

## 1 Introduction

Glaciers are increasingly known to play an active role in mobilizing the nutrients silicon (Si; Hatton, Hendry, Hawkings, Wadham, Kohler, et al., 2019) and iron (Fe; Bhatia et al., 2013; Hawkings et al., 2014) to downstream marine systems through subglacial weathering of silicate bedrock. The potential impacts on downstream nutrient cycling and marine productivity from the ongoing rapid retreat of many glaciers have thus been a focus of intense discussion. In particular, a widely debated topic involves the fate of glacially‐derived Si and Fe, focusing on the degree to which these nutrients are retained in fjords, instead of being transported to the coastal ocean to fuel more distal primary production (Hopwood et al., 2020). Addressing this question requires an improved understanding of the dissolved and particulate cycling of these nutrients in fjord settings, especially of the understudied exchange between fjord sediments and the overlying water column (benthic cycling).

Glacier‐derived bioavailable or highly reactive forms of Si include silicic acid, DSi, and dissolvable amorphous silica, ASi (Hatton, Hendry, Hawkings, Wadham, Kohler, et al., 2019; Hawkings et al., 2017). Glacier‐derived bioavailable forms of Fe include operationally defined dissolved Fe, commonly <0.2/0.45 μm or dFe, and highly reactive particulate Fe (oxy)hydroxides, hereafter termed pFe (Bhatia et al., 2013; Hawkings et al., 2014). These Si and Fe phases may be directly or indirectly utilized by phytoplankton in fjords (Meire et al., 2016; Pan et al., 2020). The sinking of phytoplankton remains (including diatom biogenic silica or BSi) and particulate mineral phases (ASi and pFe) delivered into the fjords via turbid surface meltwater runoff, subglacial plumes, and iceberg rafting, serves as pathways for glacially‐sourced Si and Fe to be deposited onto fjord bottom sediments (Hatton, Hendry, Hawkings, Wadham, Opfergelt, et al., 2019; Henkel et al., 2018; Laufer‐Meiser et al., 2021; Wehrmann et al., 2014).

Early diagenetic processes in sediments affect the solubility of reactive particulate silicon (BSi, ASi) and iron (pFe) phases. In general, BSi and ASi are subject to undersaturation and dissolution in marine pore waters (Aller, 2014). The solubility of Si can be regulated by the incorporation of aluminum (Al) and Fe into the amorphous silica structure (Aller, 2014; Van Cappellen et al., 2002), and reverse weathering—the formation of authigenic aluminosilicates or clay minerals (Ehlert et al., 2016; Michalopoulos & Aller, 2004). In addition, sediment cycling of Fe can influence pore water DSi through adsorption onto/desorption from Fe (oxy)hydroxides and co‐precipitation of amorphous Fe‐Si minerals (Delstanche et al., 2009; Geilert, Grasse, Doering, et al., 2020; Schulz et al., 2022; Zheng et al., 2016). Redox state is the main control of Fe availability in pore waters (Aller, 2014). Dissolved Fe^2+^ is released into pore waters through microbially mediated and abiotic reduction of Fe(III)‐rich minerals (Herbert et al., 2021; Laufer‐Meiser et al., 2021). Precipitation of the dissolved Fe^2+^ can also occur through oxidative formation of Fe (oxy)hydroxides in the sediments (Herbert et al., 2021; Laufer‐Meiser et al., 2021), and formation of pyrite in anoxic, sulfidic sediments (Aller, 2014).

The balance between competing diagenetic processes in fjord sediments governs not only elemental solubility but also pore water gradients, which drive the flux of these nutrients back to the overlying water column (benthic flux) via diffusion, pore water advection, bioturbation, and other sediment mixing processes (Schulz & Zabel, 2006). DSi and dFe released from the sediments may then be transported to sustain fjord, coastal and marine primary production and ecosystems via a combination of upwelling, diffusion, bottom‐water flushing, and shelf‐fjord exchange (Bianchi et al., 2020; Hopwood et al., 2020). Determining the relative magnitudes of these benthic nutrient fluxes is also important to evaluate the modification of downstream nutrient ratios and phytoplankton composition (Hopwood et al., 2020).

Dissolved stable silicon (δ^30^Si) and iron isotope (δ^56^Fe) compositions in pore waters are increasingly being employed to examine the benthic cycling of these elements, as diagenetic processes induce isotopic fractionation. For example, dissolution of glacially‐derived ASi (δ^30^Si of −0.5 to −0.2‰; Hatton, Hendry, Hawkings, Wadham, Kohler, et al., 2019; Hawkings et al., 2018) and diatom BSi (δ^30^Si ∼1–2‰ lower than ambient surface water; Frings et al., 2016) in fjord sediments are likely to impart a distinctive δ^30^Si signal to pore waters. In addition, lighter Si isotopes can be preferentially incorporated into authigenic clays (including Fe‐rich aluminosilicates), Fe (oxy)hydroxides, and amorphous Fe‐Si precipitates (Delstanche et al., 2009; Geilert, Grasse, Doering, et al., 2020; Pickering et al., 2020; Zheng et al., 2016), leaving a higher δ^30^Si signal in the pore waters. Within the ferruginous zone of marine sediments, microbially‐mediated dissimilatory Fe reduction produces Fe^2+^ that has a lighter Fe isotope composition (δ^56^Fe of −0.3 to −3‰; Crosby et al., 2007; Homoky et al., 2009; Severmann et al., 2006) than the initial Fe (oxy)hydroxide phase or crustal minerals/bulk sediment (∼+0.1‰; Beard et al., 2003). Furthermore, at the interface between the ferruginous zone and oxic surface sediments, oxidative precipitation is thought to drive the δ^56^Fe signature of upward diffusing Fe^2+^ to even lower δ^56^Fe values (Severmann et al., 2006). Lastly, in oxygenated pore waters colloidal‐phase Fe with a δ^56^Fe signature of ∼+0.1‰ could be mobilized, while ligand‐binding (commonly higher δ^56^Fe) and pyrite oxidation may also influence the δ^56^Fe signature of dFe released to bottom waters (Henkel et al., 2018; Homoky et al., 2021).

Here, we present a range of geochemical analyses, including dissolved δ^30^Si and δ^56^Fe of pore water samples from four sediment cores that were acquired from the head of Baker‐Martínez Fjord Complex (BMFC) in Chilean Patagonia. A reaction‐transport model is employed to further evaluate the complex interplay between the competing early diagenetic activities for Si.

## 2 Materials and Methods

### 2.1 Study Sites

**Figure 1:**
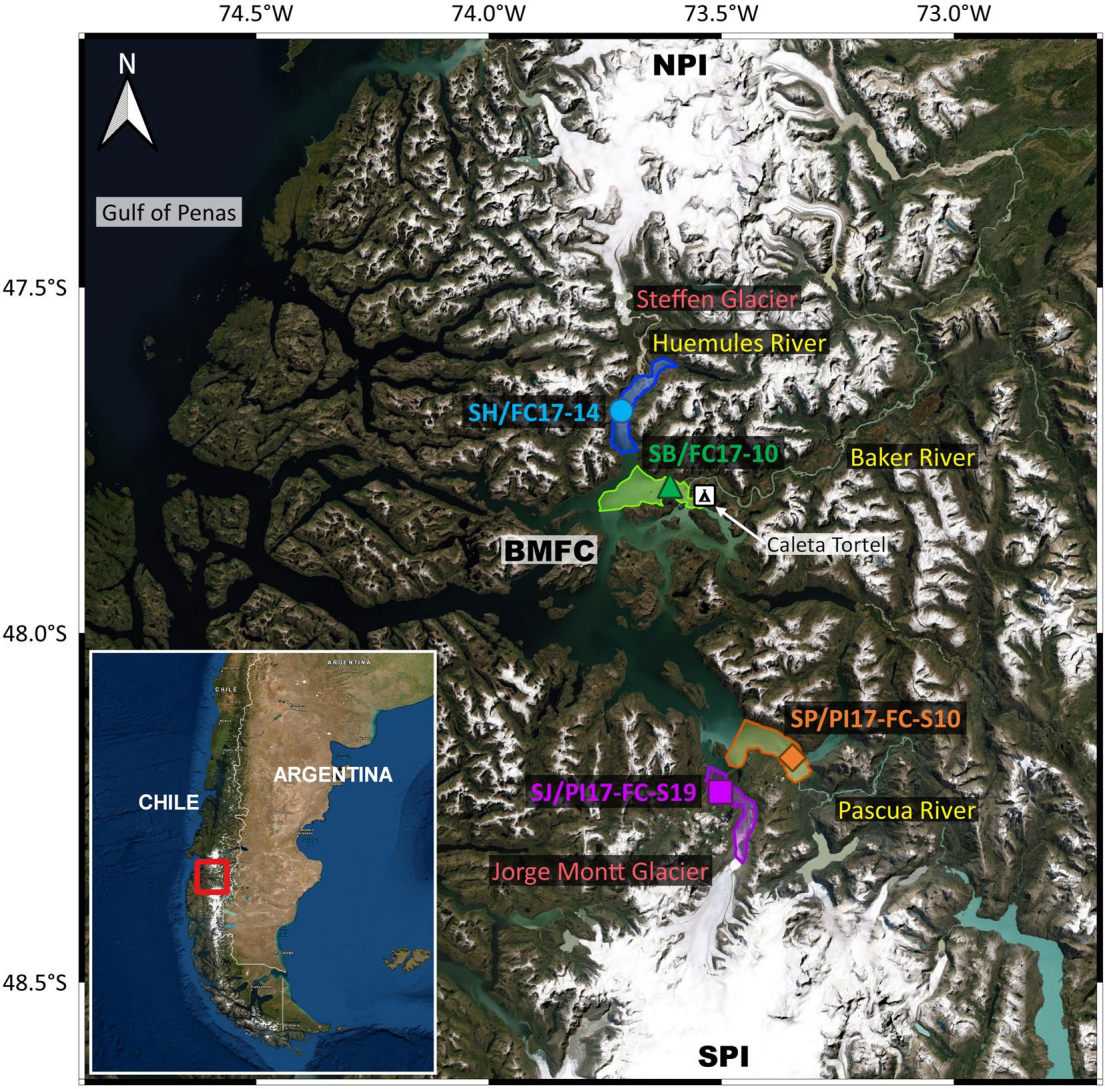
Site map. Colored symbols on the main map show the locations of the sediment core sites at the fjord heads, which are marked by the colored polygons. The bold red box on the inset map shows the extent of the main map. The figure was made using QGIS software (QGIS, 2020) and ESRI satellite imagery (Esri, 2020). NPI—Northern Patagonian Icefield, SPI—Southern Patagonian Icefield, BMFC—Baker‐Martínez Fjord Complex.

The BMFC is located between the Northern and Southern Patagonian Icefields (NPI and SPI) and is connected to the Gulf of Penas and the Pacific Ocean to the west (Figure 1). The BMFC generally has a two‐layered water mass structure: the upper layer (top 20–30 m depth) consists of estuarine water that is formed from a mix of continental freshwater and seawater, while the lower layer is composed of Subantarctic water that flows in from the Pacific Ocean through the Gulf of Penas (Quiroga et al., 2016). The major freshwater and terrestrial inputs into the fjord complex are the Baker River, the Huemules River, the Pascua River, and Jorge Montt Glacier (Troch et al., 2021). Glacier meltwater is a significant contributor to the runoff of these rivers (Amann et al., 2022; Pryer, Hawkings, et al., 2020).

The NPI sits atop the Patagonian batholith—a series of intrusive igneous rocks with a range of magmatic compositions from gabbro to granite. These igneous rocks formed from the subduction of the Antarctic plate beneath the South American plate between the Late Jurassic and the Neogene (Hervé et al., 2007). The SPI is predominantly underlain by the Eastern Andes Metamorphic Complex, which is mainly composed of Paleozoic greenschist‐facies metasediments (Hervé et al., 2008). Subglacial weathering of these igneous and metamorphic bedrocks produces high concentrations of colloidal and reactive particulate Si and Fe phases (Pryer, Hawkings, et al., 2020), which are delivered into the BMFC.

### 2.2 Field Sampling and Sediment Cores

Bottom water temperature, salinity, and dissolved oxygen concentration measurements at or near the core sites were acquired using an RBR maestro conductivity‐temperature‐depth (CTD) sensor. A gravity corer fitted with 1.5 m long transparent core liners was used to acquire sediment cores from four sites in the BMFC (Figure 1) on the research vessel Sur‐Austral in February 2017. The equipment available on the research vessel was suited for relatively shallow coring operations (<300 m depth), and hence sediment cores were only collected from the fjord heads. The four core sites (Table S1 in Supporting Information S1): SJ (106 m depth), SH (203 m depth), SP (248 m depth) and SB (151 m depth) were situated close to the outflows of the Jorge Montt Glacier, Huemules River, Pascua River, and Baker River, respectively. One set of sediment cores collected from these four sites was used for sampling filtered core‐top water, followed by sampling of filtered pore water, using syringes attached with Rhizon pore water samplers (0.15 μm; Rhizosphere) that were inserted through holes pre‐drilled on the core liners (Ng et al., 2020). The collected water samples were acidified by adding 1% v/v 10 M lab distilled HCl prior to storage.

Another set of cores was obtained from the four fjord sites for sediment analysis (Table S1 in Supporting Information S1). Sediment physical properties such as porosity and bulk density were measured on this set of cores using Gamma ray attenuation on a Geotek Multi‐Sensor Core Logger, prior to subsampling of solid sediments for subsequent analyses. Sedimentation rate(s) of core SJ was based on annual variations in ice‐rafted debris deposition (De Wilde, 2016), cores SH and SP were based on ^137^Cs (Piret et al., 2021, 2022), and core SB was based on ^210^Pb (Vandekerkhove et al., 2021).

### 2.3 Dissolved Nutrient and Element Concentration Measurements

Pore water and core‐top water dissolved Si and nitrate concentrations, [DSi] and [NO_3_^−^] respectively, were analyzed in the LOWTEX facility at the University of Bristol. Sample [DSi] was measured with the molybdate‐blue method (DeMaster, 1981) using the LaChat 8500 Series‐2 flow injection analyzer. [NO_3_^−^] was measured using a Thermo Gallery discrete analyzer after samples were reduced with vanadium chloride and reacted with the Griess reagent (Wang et al., 2016). The analytical errors of the methods were ∼2.5% for [DSi] and ∼0.5% for [NO_3_^−^], derived from 2 SD (standard deviation) of replicate measurements of samples and TraceCERT^®^ standards.

Concentrations of dissolved (<0.15 μm) iron [dFe] and manganese [dMn] were measured using a Thermo Scientific X‐Series 2 quadrupole inductively coupled plasma‐mass spectrometer (ICP‐MS) with a collision/reaction cell at the National Oceanography Centre, Southampton, after a ×30 gravimetric dilution in 2% v/v HNO_3_. Beryllium, indium and rhenium were used as internal standards to correct for drift and matrix effects. Multi‐element external calibration solutions were gravimetrically made to match the concentration range observed in samples. All measurements were above the instrument's limit of detection (∼3 nM for Fe, ∼0.1 nM for Mn). Measurement accuracy was determined by analyzing the gravimetric multi‐element external calibration solutions, and precision was determined by repeat measurements on the gravimetric calibration solutions of intermediate concentrations. Analytical precision for the elements analyzed ranged from ±0.7 to ±8.9%, and accuracy was better than ±10%.

### 2.4 Fe Isotope Analysis

Aliquots of the pore water and core‐top water samples were used for Fe isotope analysis at the University of South Florida (USF). All work was carried out under ISO‐5 clean conditions, all water was 18.2 MΩ. cm from a Genpure Barnstead System and all acids were single‐distilled from trace metal grade acids via Savillex™ DST‐1000 stills. A ^57^Fe‐^58^Fe double spike was added to samples in a sample‐to‐spike ratio of 1:2. Samples were dried down, redissolved and refluxed in concentrated HNO_3_ with 10% H_2_O_2_ (Fisher Ultrapure grade) to digest any organic material. Samples were then processed using an AGMP‐1 resin microcolumn purification scheme following Conway et al. (2013), as modified by Sieber et al. (2021). Following purification, samples were dried down and then reconstituted in 2% v/v HNO_3_ for instrumental analysis. The method has a total procedural blank of <0.4 ng (Sieber et al., 2021).

Iron isotope ratios were measured using a Thermo Neptune MC‐ICP‐MS in “high” resolution mode at the USF Tampa Bay Plasma Facility, using a ∼100 μL min^−1^ PFA nebulizer and an ESI Apex Ω introduction system. Analysis proceeded as described in Sieber et al. (2021); beams were corrected for background using an on‐peak measurement of 2% v/v HNO_3_ immediately prior to each sample, the abundances of ^53^Cr and ^60^Ni were measured to correct ^54^Cr and ^58^Ni isobaric interferences on ^54^Fe and ^58^Fe, and correction for instrumental mass bias was carried out using the double spike method (Siebert et al., 2001). Sample δ^56^Fe are expressed relative to the mean of two IRMM014‐double spike bracketing standards (per block of 6 samples). At USF the long‐term 2 SD of analysis (±0.04‰) of NIST‐3126a is used as a conservative estimate of analytical precision (Sieber et al., 2021). We therefore express uncertainty on δ^56^Fe measurements as 0.04‰, or the 2 SE (standard error) of an individual measurement when >0.04‰.

### 2.5 Analysis of Sediment Reactive Si Phases

Operationally‐defined reactive Si phases in the sediments analyzed here are (a) mild acid‐leachable silica (Si‐HCl) associated with Fe, Mn and Al oxides (Michalopoulos & Aller, 2004; Pickering et al., 2020), and (b) mild alkaline‐leachable amorphous silica (Si‐Alk) that includes BSi and glacier‐derived ASi (Ng et al., 2020; Pryer, Hatton, et al., 2020). Analyses of these phases were carried out using sequential extraction, with procedures modified from Michalopoulos and Aller (2004), Pickering et al. (2020), and Ng et al. (2020).

Si‐HCl was extracted from ∼50 to 100 mg of air‐dried sediments by treating the samples with 36 mL of 0.1 M HCl (lab distilled) and shaking for 18 hr. Samples were centrifuged, and the supernatant was filtered through 0.2 μm Sartorius™ polyethersulfone (PES) syringe filters. The pH of the Si‐HCl leachates was adjusted to ∼5 using 4 M NaOH (Honeywell Fluka™; brown Fe precipitates would form at pH 7) before the leachates were analyzed for [DSi] and the Si isotopic composition. The remaining sediments were rinsed with 18.2 MΩ.cm Milli‐Q water, treated with 5 mL of 10% H_2_O_2_ (Romil SpA™) for 0.5 hr to remove organic matter, and rinsed again with Milli‐Q water.

Si‐Alk was extracted from the HCl‐ and H_2_O_2_‐treated sediments by digesting the sediment in 40 mL of 0.1 M Na_2_CO_3_ (ACROS Organics™) in a water bath at 85°C (DeMaster, 1981). A portion of the leachate was taken 20 min into the digestion for Si isotope analysis before a significant amount of lithogenic material started to dissolve. Aliquots were also taken at 2, 3, and 5 hr to quantitatively distinguish the rapidly dissolving BSi and ASi (Si‐Alk) from the slowly released lithogenic silica. Leachates were filtered through 0.2 μm syringe filters (as above) and neutralized with HCl prior to instrumental analysis. A linear regression was fitted through the measured leachate Si content at 2, 3, 5 hr of digestion, and the intercept at 0 hr was used to quantify the Si‐Alk concentration.

Si content in the acid and alkaline leachates were measured with the molybdate‐blue method (DeMaster, 1981) using a V‐1200 Vis spectrophotometer (Ng et al., 2020). Procedural blanks were below the detection limit (<0.01 μM). Uncertainties in the sequential extraction procedures were calculated using average standard deviations of repeat sediment leaches and measurements of Si content in the Si‐HCl and Si‐Alk phases, as ±15% and ±25%, respectively.

### 2.6 Si Isotope Analysis

For the pore water and core‐top water samples, pre‐concentration of Si was carried out using a Mg‐induced co‐precipitation method (de Souza et al., 2012). The method involved precipitation of Mg(OH)_2_ with 1 M NaOH (Titripur^®^Reag. Ph Eur grade), followed by rinses of 0.001 M NaOH to quantitatively remove excess major ions: Na^+^, Cl^−^, SO_4_^2−^, Ca^2+^, and K^+^, after which the Si‐containing precipitates were dissolved with HCl (lab distilled) to a smaller volume. Using the spectrophotometer (Section 2.5), aliquots of the concentrated samples were measured for [DSi] to monitor the yields of the co‐precipitation method, which were >95%.

The pre‐concentrated pore water, core‐top water samples and the sediment leachate samples were further purified with cation exchange column chemistry following the procedures of Georg et al. (2006). The column chemistry consisted of polypropylene columns containing 1.8 mL Bio‐Rad AG50W‐X12, 200–400 mesh cation exchange resin in H^+^ form, which was pre‐rinsed with HCl and Milli‐Q before the samples were loaded onto the columns and eluted with ultra‐pure water.

Sample solutions were introduced into a Thermo‐Finnigan Neptune MC‐ICP‐MS at the Bristol Isotope Group laboratory using a CETAC PFA spray chamber (wet plasma) and PFA nebulizer (∼100 μl min^−1^). The method of analysis for Si isotopes followed Ng et al. (2020). Briefly, sample, bracketing standard (NBS‐28) and reference standard (Diatomite, LMG08, ALOHA_1000_) solutions were doped with H_2_SO_4_ (ROMIL UpA™) and HCl (lab distilled) to alleviate the anionic matrix effects (Hughes et al., 2011). Instrumental mass bias and matrix effects were corrected using standard‐sample bracketing and Mg‐doping methods (Cardinal et al., 2003). Data quality was monitored through replicate analyses of several reference standards. Measurements of Diatomite, LMG08 sponge, and ALOHA_1000_ seawater standards yielded δ^30^Si of +1.24 ± 0.08‰ (*n* = 18), −3.47 ± 0.10‰ (*n* = 25), and +1.24 ± 0.08‰ (*n* = 8), respectively, which agreed with published values within 2 SD (Grasse et al., 2017; Hendry & Robinson, 2012; Reynolds et al., 2007). The δ^29^Si and δ^30^Si values of all standards and samples measured fell on the mass‐dependent fractionation line with a gradient of 0.5152 ± 0.0031, lying between thermodynamic (0.5210) and kinetic (0.5105) values (Cardinal et al., 2003). Typical analytical uncertainties were evaluated based on 2 SD of sample replicate measurements (*n* = 2–3), which ranged between 0.12 and 0.34‰ for water samples, and 0.001–0.21‰ for sediment leachates. Such replicate analysis was only carried out when there was sufficient sample available.

### 2.7 Reaction‐Transport Modeling for Si

The concentration and isotope profiles of dissolved and solid Si phases in the fjord sediments were simulated using the Biogeochemical Reaction Network Simulator (BRNS), an adaptive simulation environment for mixed kinetic‐equilibrium reaction networks (Aguilera et al., 2005; Regnier et al., 2002). Our BRNS design followed the model setup detailed in Cassarino et al. (2020) and Ward et al. (2022a). We assumed that Si diagenetic processes were at steady state. Although fjord systems are subject to transient episodes of glacial runoff, phytoplankton blooms and sediment deposition (Bianchi et al., 2020), the assumption of steady state allows us to define the long‐term dynamics within fjord sediments and offers a first‐order estimate of the diagenetic processes governing the benthic Si cycle at fjord heads.

The model is based on a vertically‐resolved reaction‐transport mass conservation equation that computes concentration changes of dissolved and solid phases in porous media (Berner, 1980; Boudreau, 1997):

(1) $$\frac{\partial \sigma C_{Si}}{\partial t}=\frac{\partial }{\partial z}\left[\left(D_{bio}+D_{m\_Si}\right)\frac{\partial \sigma C_{Si}}{\partial z}\right]-\frac{\partial \sigma \omega C_{Si}}{\partial z}+\alpha_{irr}\sigma \left[C_{Si}\left(0\right)-C_{Si}\right]+\sum_{j}\lambda_{Si,\;j}R_{j}$$

*t*, *z*, *C*_Si_ are time, depth, and concentration of Si respectively. *ω* denotes the measured sedimentation rate (Table S2 in Supporting Information S1). For the dissolved phase: *σ* = porosity or *ϕ*, while for the solid phase: *σ* = (1 – *ϕ*). The model assumed an exponential decrease in porosity due to sediment compaction (e.g., Freitas et al., 2020), which was calculated based on the measured core‐top porosity, *ϕ*_0_, measured bottom porosity, *ϕ*_*x*_, and a fitted porosity attenuation coefficient, *β*_*ϕ*_ (Table S2 in Supporting Information S1). Sediment bioturbation was modeled as a diffusive‐like process, constrained with the bioturbation coefficient, *D*_bio_ and bioturbation depth, *z*_bio_ (Table S2 in Supporting Information S1). Values of *D*_bio_ and *z*_bio_ were inferred from a benthic macrofauna study in fjords (Oleszczuk et al., 2019). The coefficient of molecular diffusion of DSi, *D*_*m_*Si_ (*D*_*m_*Si_ = 0 for solids) was derived based on an empirical relationship (Rebreanu et al., 2008) with measured temperature, *T* and measured salinity, *S* (Table S2 in Supporting Information S1). Bioirrigation rate, *α*_irr_ (*α*_irr_ = 0 for solids) was computed as a function of the bioirrigation coefficient, *α*_0_ and bioirrigation attenuation depth, *x*_irr_ (Table S2 in Supporting Information S1). *α*_0_ was inferred from the benthic macrofauna study in fjords (Oleszczuk et al., 2019), while a global mean value (Freitas et al., 2020; Ward et al., 2022a) was assumed for *x*_irr_ (Table S2 in Supporting Information S1).

The final term of Equation 1 represents the sum of reactions *j* affecting the Si phases, with reaction stoichiometric coefficient *λ*_*Si*,*j*_ and rate *R*_*j*_. We implemented a reaction network that resolved the dissolution of an undersaturated Si‐Alk phase (BSi, glacial ASi) and the precipitation of authigenic aluminosilicate clay minerals (AuSi) associated with reverse weathering. The kinetic rate laws associated with the dissolution and precipitation reactions, *R*_*d*_ and *R*_*p*_ respectively, are expressed as:

(2) $$R_{d}=k_{diss}\cdot \exp\left(ad\cdot z+bd\right)\cdot \left[Si-Alk\right]\cdot \left(1-\frac{\left[DSi\right]}{{\left[ASi\right]}_{sat}}\right)\;if\;\left[DSi\right]<\;{\left[ASi\right]}_{sat}$$

(3) $$R_{p}=k_{precip}\cdot \exp\left(ap\cdot z+bp\right)\cdot \left(\frac{\left[DSi\right]}{{\left[AuSi\right]}_{sat}\;}-1\right)\;if\;\left[DSi\right]>\;{\left[AuSi\right]}_{sat}$$

*k*_diss_ and *k*_precip_ are fitted rate constants. *ad*, *bd*, *ap*, and *bp* are fitted coefficients defining the exponential decreases of the dissolution and precipitation reactions (Cassarino et al., 2020; Ehlert et al., 2016; Ward et al., 2022a). [Si‐Alk] represents the sediment concentration of amorphous silica (BSi, glacial ASi). [ASi]_sat_ and [AuSi]_sat_ are the saturation concentrations of the amorphous silica and the authigenic aluminosilicates, respectively, based on values derived from previous studies (Table S2 in Supporting Information S1). We assume an exponential decrease of *R*_*d*_ (Equation 2) to account for the rapid decline of amorphous silica solubility due to the incorporation of Al and Fe in the sediments (Aller, 2014; Van Cappellen et al., 2002). Similarly, we assume that *R*_*p*_ also decreases exponentially (Equation 3), following observations that authigenic clay precipitation occurs predominantly in the upper sediments (Michalopoulos & Aller, 2004), potentially due to the greater availability of reactive Al and Fe phases.

The core‐top concentrations and isotope compositions of DSi, Si‐Alk, and Si‐HCl measured in Section 2.5 and 2.6 were utilized to define the upper boundary conditions of the BRNS‐Si model. The δ^30^Si measurements from Section 2.6 were converted to explicit ^28^Si and ^30^Si isotope concentrations for the model simulation through a series of isotope mass balance equations (Equation S1–S4 in Supporting Information S1; Cassarino et al., 2020; Ward et al., 2022a). The model assumes that the total abundance of Si is represented by the sum of ^28^Si and ^30^Si (^29^Si not included), and so there is an error of under 5% associated with the simulated concentration of a given Si phase. Potential isotopic fractionation associated with the dissolution and precipitation reactions are accounted for by the fractionation factors *α*_*d*_ and *α*_*p*_ respectively (Equation S5–S8 in Supporting Information S1), based on values derived from previous studies (Table S2 in Supporting Information S1).

Our understanding of coupled Si‐Fe cycling in marine sediments is relatively new. Detailed mathematical formulation of the associated reactions requires future experimental studies and more observational data, especially on the fraction of Si adsorbed onto or co‐precipitated with Fe(III) phases in seawater and pore water composition. Here, simplified constant reaction rates (Ward et al., 2022a) were used to simulate Si‐Fe reactions. *R*_up28_ and *R*_up30_ are model‐optimized constant rates of uptake of pore water ^28^Si and ^30^Si by reactive Fe phases through adsorption and co‐precipitation in the relatively oxic upper sediment layer. In contrast, *R*_re28_ and *R*_re30_ are model‐optimized constant rates of release of ^28^Si and ^30^Si into pore water from reductive dissolution of Fe in the ferruginous zone (Table S2 in Supporting Information S1). The upper and lower depth limits of the oxidizing layer, *z*_ox‐up_ and *z*_ox‐low_, and those of the ferruginous zone, *z*_fe‐up_ and *z*_fe‐low_ (Table S2 in Supporting Information S1), were inferred from measured pore water dFe concentration profiles. The sediment Si‐HCl phase is thought to be partly composed of Si associated with metal oxides including Fe oxides (Pickering et al., 2020). Therefore, in the model we allowed sediment Si‐HCl content and its isotope composition to vary following the modeled *R*_up28_, *R*_up30_, *R*_re28_, and *R*_re30_ (Equation S9–S12 in Supporting Information S1).

The length of the model domain, *L*_*M*_, was set based on the maximum depth where pore water DSi data were available (Table S2 in Supporting Information S1). The model run time was set to allow for two full sediment deposition cycles (runtime = 2 × *L*_*M*_ ÷ *ω*), thus ensuring that steady state was achieved. The model was run on an uneven depth resolution grid, which allowed for a fine resolution of the upper, most active sediment layers (e.g., Freitas et al., 2020). The depth intervals were resolved at 0.1 cm interval from 0 to 3 cm depth, at 0.2 cm interval from 3 to 5 cm depth, at 0.4 cm interval from 5 to 10 cm depth, at 0.6 cm interval from 10 to 20 cm depth, and at 1 cm interval for sediment depths greater than 20 cm.

We ran model ensembles until a best‐fit solution was found. The best‐fit solution was determined by minimizing the misfit (specifically, the root‐mean‐square error or RMSE: Equation S13 and S14 in Supporting Information S1) between the measured and modeled depth profiles of Si concentration and isotope composition (Table S3 in Supporting Information S1). Our model's outputs include rates of reaction *R*_*d*_ and *R*_*p*_, as well as the concentrations and isotope compositions of pore water DSi, sediment Si‐Alk and Si‐HCl. As such, the model allows us to quantify parameters defining the rates of Si‐Alk dissolution (*k*_*diss*_, *ad* and *bd*), AuSi precipitation (*k*_*precip*_, *ap* and *bp*), and coupled Si‐Fe cycling (*R*_up28_, *R*_up30_, *R*_re28_, and *R*_re30_).

## 3 Results and Discussion

### 3.1 Redox Conditions and Pore Water dFe in BMFC

**Figure 2:**
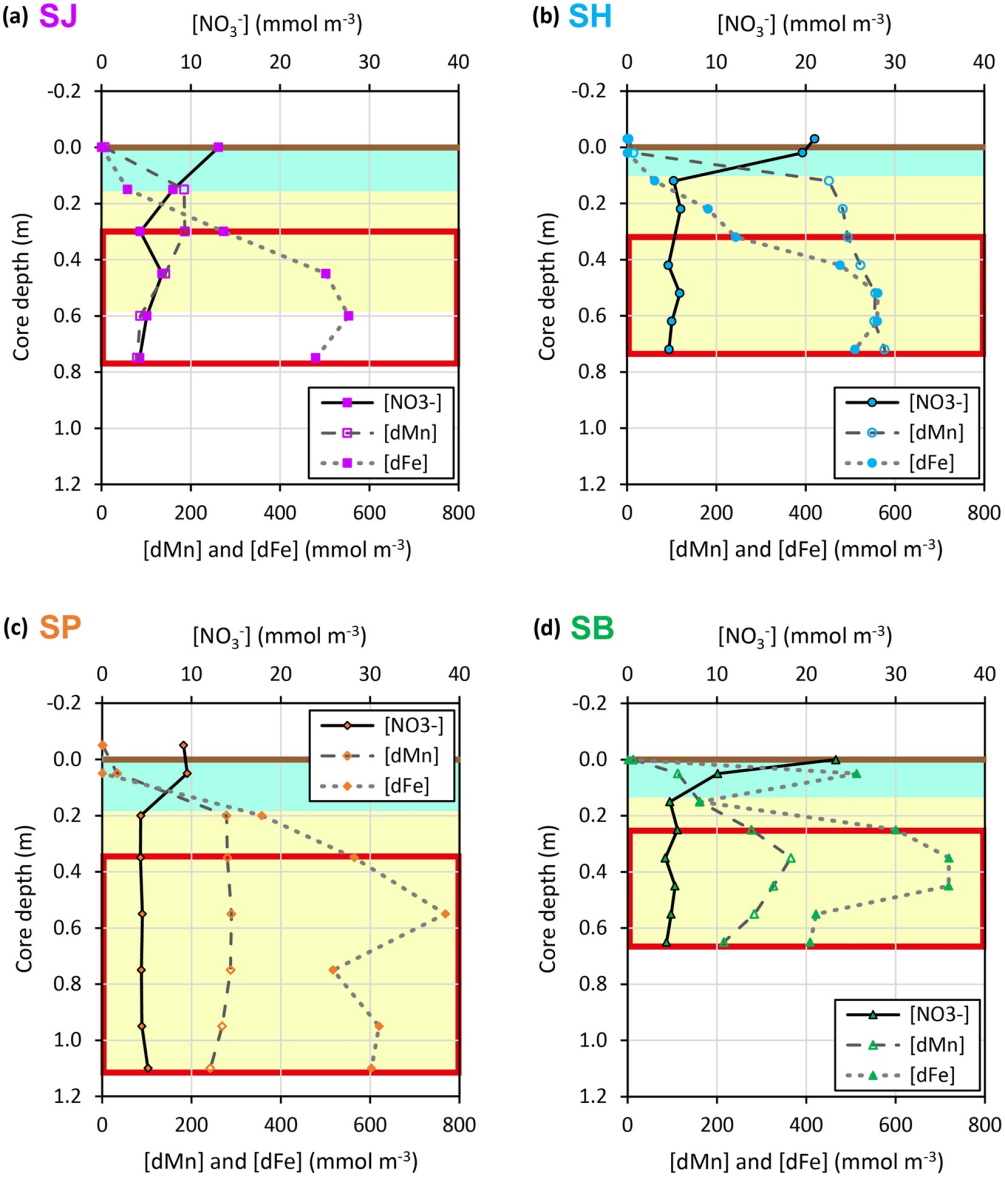
Dissolved concentrations of redox‐sensitive elements: [NO_3_^−^], [dMn], and [dFe]. Results for sites (a) SJ, (b) SH, (c) SP, and (d) SB. The horizontal brown line marks the sediment‐water interface. Turquoise shading, yellow shading, and the red box indicate the sediment cores' nitrogenous, manganous, and ferruginous zones respectively.

**Figure 3:**
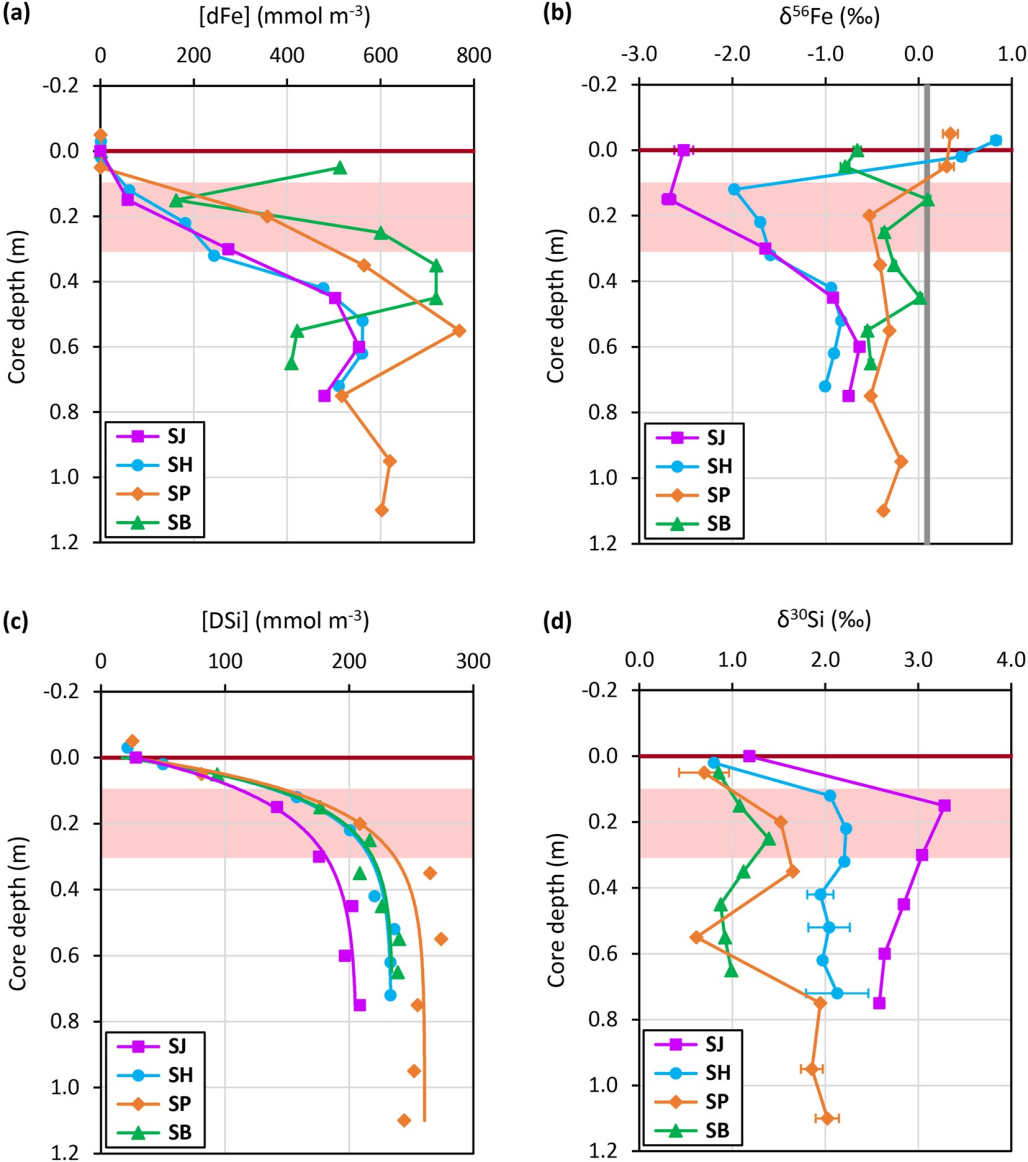
Pore water and core‐top water Fe and Si results. (a) [dFe], (b) δ^56^Fe, (c) [DSi], and (d) δ^30^Si profiles. The horizontal brown lines mark the sediment‐water interface. The red shading broadly highlights the Fe(II)‐Fe(III) redox boundary inferred from the pore water profiles of redox‐sensitive elements (Figure 2). The thick vertical gray line indicates the average crustal δ^56^Fe value (Beard et al., 2003). Lines in the [DSi] plots are least squares exponential fitting of the data carried out using SigmaPlot (Systat Software Inc.). Error bars represent 95% confidence intervals of δ^56^Fe and δ^30^Si measurements, and some are smaller than the symbols.

Bottom water dissolved oxygen concentrations at the fjord heads (160–180 mmol m^−3^) and [NO_3_^−^] in the core‐top water samples (Figure 2) indicate the presence of oxygenated bottom water and surface sediments at all four study sites. The oxygenated core‐top waters exhibit [dFe] that range from 0.3 to 1.2 mmol m^−3^. Sediments turn suboxic below ∼0.1 m core depth, where rapid depletion of [NO_3_^−^] and substantial increases of [dMn] and [dFe] are observed in the pore water (Figures 2 and 3a). In particular, the ferruginous zones at all the study sites (Figure 2) are deeper, broader, and with higher maxima in pore water [dFe] (550–770 mmol m^−3^) than other coastal marine and open ocean sediments observed, but similar to selected sites from glaciated fjords in Svalbard (Herbert et al., 2020, 2021, 2022; Laufer‐Meiser et al., 2021; Wehrmann et al., 2014, 2017). The broader ferruginous zones and [dFe] maxima at our BMFC sites are most likely due to a high supply of reactive Fe phases from glacier‐fed rivers (Pryer, Hawkings, et al., 2020), land‐terminating and marine‐terminating glaciers (Henkel et al., 2018; Wehrmann et al., 2014).

In all BMFC cores, pore water δ^56^Fe at depths below 0.5 m cluster around −1 to −0.2‰ (Figure 3b), which are lower than the average crustal δ^56^Fe value (∼+0.1‰; Beard et al., 2003). These lower δ^56^Fe values most likely reflect an imprint of microbially mediated dissimilatory Fe reduction within the ferruginous zone (Crosby et al., 2007; Homoky et al., 2009; Severmann et al., 2006). In contrast, there is a notable difference in the shallower pore water δ^56^Fe profiles between the BMFC sites (Figure 3b), which is likely the result of multiple processes that involve lithogenic colloids/nanoparticles, organic ligands, and redox‐driven dissolution and re‐precipitation of Fe. Fe isotope signatures close to or heavier than crustal compositions (+0.3 to +0.8‰) are observed in both the shallowest, oxic pore waters (<0.1 m depth) and the overlying core‐top waters of SH and SP sites. The heavier Fe isotope signatures potentially reflect isotopic fractionation by “non‐reductive” formation of lithogenic Fe(III) colloids/nanoparticles (<0.15 μm; Radic et al., 2011; Homoky et al., 2021), or by the binding of Fe(III) to organic ligands (Dideriksen et al., 2008; Morgan et al., 2010), that mainly occur in oxygenated waters.

### 3.2 Spatial Differences in Fe Redox Cycling

Sites SJ and SH show pore water δ^56^Fe minima (−2–−3‰) at the Fe(II)‐Fe(III) redox boundary (just above the ferruginous zone, Figure 2) around 0.1–0.3 m depth (Figure 3b). These isotopically‐light Fe compositions can be explained by intense Fe redox cycling that involves: **[i]** reductive dissolution of Fe yielding isotopically light Fe^2+^ (Crosby et al., 2007) in the ferruginous zone, **[ii]** upward advection and diffusion of Fe^2+^ to the relatively oxic sediment layer, **[iii]** oxidation of Fe^2+^ to amorphous Fe(III) (oxy)hydroxides (Homoky et al., 2009) driving the remaining Fe^2+^ even lighter (Severmann et al., 2006). In contrast, the lack of an evident pore water δ^56^Fe minimum around the Fe(II)‐Fe(III) redox boundary (∼0.1–0.3 m depth) in the other two cores (Figure 3b) suggests that the Fe redox cycling described above occurs to a far lesser extent at the SP and SB sites.

Here, we examine the potential factors that could be responsible for the observed spatial differences in the intensity of Fe redox cycling. The intensity of Fe(III) reduction (step **[i]**) is partly linked to the supply of easily reducible Fe (oxy)hydroxides, such as ferrihydrites, which differ between the fjord sites. The Huemules river upstream of the SH site (Figure 1) has been found to yield 10 times higher amounts of highly reactive Fe than the Baker and Pascua rivers upstream of the SB and SP sites respectively (Table S4 in Supporting Information S1; Pryer, Hawkings, et al., 2020). The higher Fe input to SH has been linked to the greater extent of glacial cover in the Huemules catchment (Pryer, Hawkings, et al., 2020). An even greater yield of highly reactive Fe is expected at the SJ site given the supply of glacial sediments through ice rafting and subglacial discharge without proglacial lake processing (Laufer‐Meiser et al., 2021). The greater rate of supply of highly reactive Fe available at SJ and SH is thought to sustain increased microbial Fe reduction, which could contribute to the lighter pore water δ^56^Fe signals observed at these two sites (Figure 3b).

A dFe uptake mechanism in the relatively oxic upper sediment layer involves an upward transport of Fe^2+^ from the underlying ferruginous zone and subsequent Fe^2+^ oxidation (steps **[ii]** and **[iii]** of Fe redox cycling). The intensity of the upward transport (step **[ii]**) is a function of pore water advection, which can differ substantially between the study sites depending on sediment physical properties and depositional environment. For example, given its proximity to the marine‐terminating Jorge Montt Glacier (Figure 1), the SJ site has the highest sedimentation rate and the highest sediment porosity (Table S1 in Supporting Information S1), which would maintain a significantly larger compaction‐driven, upward pore water advection (Santos et al., 2012; Schulz & Zabel, 2006) compared to the other study sites. Bottom currents flowing over the uneven surface sediments is another common mechanism maintaining pore water advection through the top several tens of centimeters of permeable sediments (Santos et al., 2012; Schulz & Zabel, 2006). The bottom current‐induced pore water advection most likely occurs at the SJ and SH sites where the upper sediments have permeable sandy layers (Table S1 in Supporting Information S1), likely sourced from direct glacial sediment inputs from Jorge Montt Glacier, and from periodic glacial lake outburst floods (GLOF) from Steffen Glacier (Piret et al., 2021), respectively. Meanwhile, bottom current‐induced pore water advection is most likely impeded at the SP and SB sites, where the upper sediments (<0.5 m, Table S1 in Supporting Information S1) are less permeable muds and silts (Santos et al., 2012; Schulz & Zabel, 2006) mainly deposited from riverine outflows (Piret et al., 2022; Vandekerkhove et al., 2021).

The spatial difference in sediment physical properties between the fjord sites likely indicates decreasing intensity of pore water advection from SJ, SH, SP to SB. Therefore, upward flow of Fe^2+^ and subsequent Fe^2+^ oxidation (step **[ii]** and **[iii]** of Fe redox cycling) are likely most intense at SJ, followed by SH, SP, and SB. The inferred trend is consistent with the pore water δ^56^Fe observation at around the Fe(II)‐Fe(III) redox boundary, where the lowest δ^56^Fe minimum is observed at SJ, followed by SH, and the lack of such δ^56^Fe minima at SP and SB (Figure 3b). In short, the intensity of Fe redox cycling at the fjord sites appears to be related to sediment physical properties, depositional environment, and the rate of supply of highly reactive Fe such as amorphous Fe (oxy)hydroxides.

### 3.3 Processes Influencing Sediment Pore Water Si in BMFC

**Figure 4:**
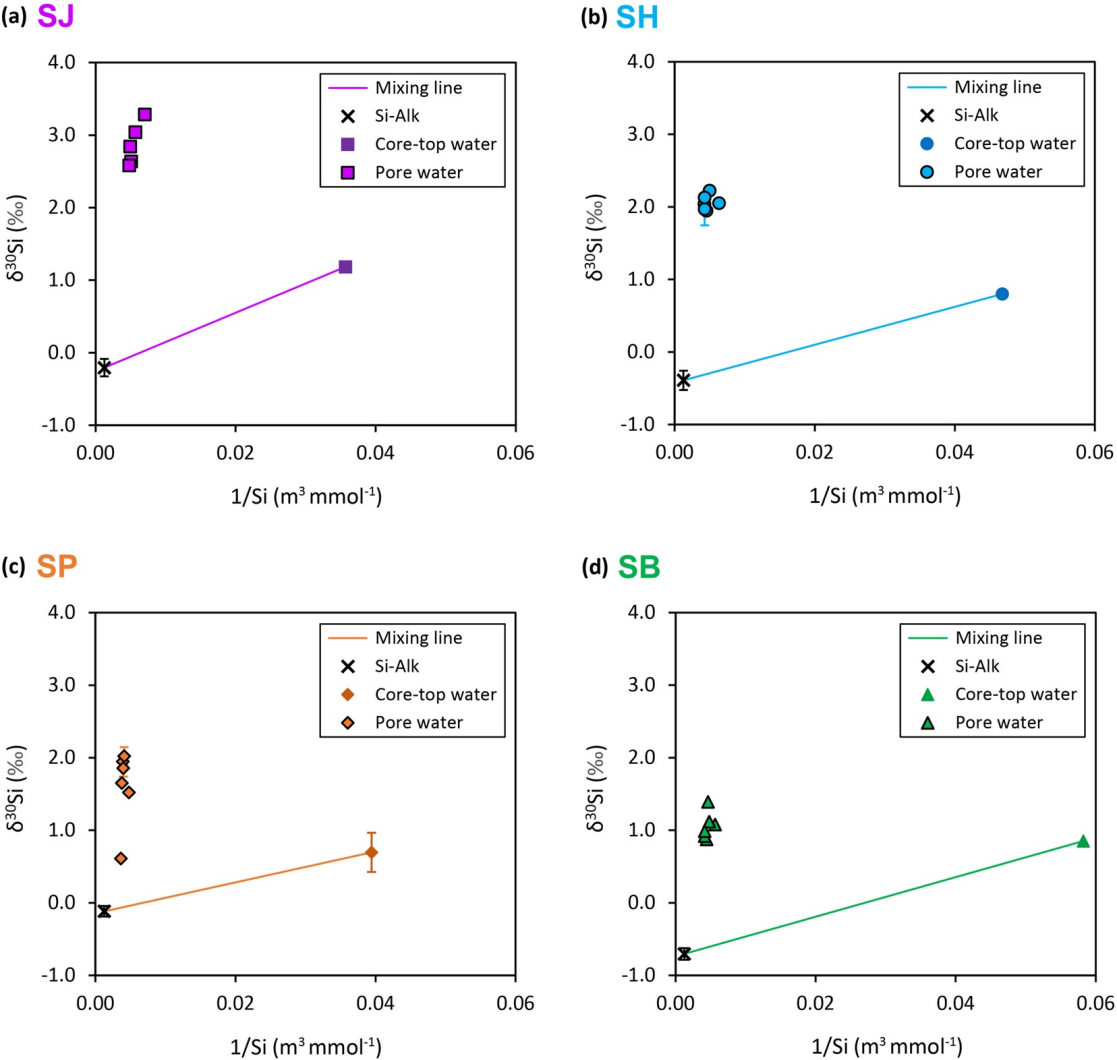
Plots of δ^30^Si against 1/[DSi] of pore water and linear mixing models between the core‐top water and Si‐Alk endmembers. Sites (a) SJ, (b) SH, (c) SP, and (d) SB. Core‐top water [DSi] for SB is extrapolated based on least‐squares exponential fitting of available pore water data (Figure 3c). Core‐top water δ^30^Si for SH, SP, and SB are assumed to be the same as the shallowest available (2–5 cm) pore water data (Figure 3d). Si‐Alk δ^30^Si are whole core averages.

The core‐top water [DSi] ranges between 21 and 26 mmol m^−3^. Pore water [DSi] at all sites exhibit asymptotic exponential profiles (Figure 3c), where [DSi] increases with increasing core depths at ever‐decreasing rates toward values ranging between ∼200 and 260 mmol m^−3^. The increase in pore water [DSi] with depth indicates a net supply of Si from the dissolution of undersaturated sediment silica phases, which are dominantly represented by Si‐Alk (BSi, glacial ASi) in the sediments (Ng et al., 2020; Pryer, Hatton, et al., 2020). For sites SJ, SH and SP, the δ^30^Si of the Si‐Alk phases fall between 0 and −0.5 ‰ (Figure 4, Figure S1 in Supporting Information S1), which is within the range of previously published glacially‐derived ASi isotope endmembers (Hatton, Hendry, Hawkings, Wadham, Kohler, et al., 2019; Hawkings et al., 2018). Lower Si‐Alk δ^30^Si is observed at the SB site (Figure 4, Figure S1 in Supporting Information S1), which suggests a greater relative abundance of other Si‐Alk phases that could have a lighter isotope composition than glacial ASi.

If Si‐Alk dissolution is the sole process governing benthic Si cycling, pore water [DSi] and δ^30^Si should lie on the mixing line between the core‐top water endmember: [DSi]_ctw_ and δ^30^Si_ctw_, and the Si‐Alk endmember. Theoretical mixing of DSi concentration, [DSi]_mix_, and its isotope composition, δ^30^Si_mix_, between the two endmembers can be calculated with the following model (Geilert, Grasse, Doering, et al., 2020):

(4) $${\left[DSi\right]}_{mix}=\left({\left[DSi\right]}_{ctw}\cdot f\right)+\left({\left[ASi\right]}_{sat}\cdot \left(1-f\right)\right)$$

(5) δSimix=30δSictw30·[DSi]ctw·f+δSi−Alk30·[ASi]sat·(1−f)[DSi]mix

[ASi]_sat_ denotes the saturation concentration of amorphous silica (Table S2 in Supporting Information S1). δ^30^Si‐Alk represents the average δ^30^Si of the Si‐Alk phase in a given core. *f* is mixing fraction, with *f* = 0 signifying 100% fluids derived from Si‐Alk dissolution, and *f* = 1 indicating 100% core‐top water.

All pore water data lie well above the linear mixing models computed for each of the sediment cores (Figure 4), providing clear evidence that benthic Si cycling at the fjord sites is regulated by other processes, in addition to Si‐Alk dissolution. Two major diagenetic processes that can account for the observation above are: (a) reverse weathering—precipitation of AuSi (Ehlert et al., 2016; Ng et al., 2020) including Fe‐rich aluminosilicates (Geilert, Grasse, Doering, et al., 2020; Isson & Planavsky, 2018); (b) interaction with Fe, including adsorption onto Fe (oxy)hydroxides and co‐precipitation of amorphous Fe‐Si minerals (Delstanche et al., 2009; Geilert, Grasse, Doering, et al., 2020; Zheng et al., 2016). These diagenetic processes preferentially remove isotopically light Si isotopes from solution and lead to high pore water δ^30^Si values, which are far above the mixing line between the core‐top water and Si‐Alk endmembers (Figure 4). AuSi precipitation and coupled Si‐Fe reactions are further examined in Section 3.4 and 3.5.

A significant drop of pore water δ^30^Si (Figure 3d) and an evident transition of sediment Si‐Alk content (Figure S1 in Supporting Information S1) are observed at 0.55 m in core SP. These changes coincide with a sharp decrease in sediment grain size above 0.55 m, which is attributed to a hydrological reorganization of the Pascua River following a GLOF event that occurred in the 1940s (Piret et al., 2022). Our data suggest that the GLOF and resultant turbidity current may have transported a significant amount of isotopically light Si phases, which explains the anomalous pore water δ^30^Si (Figure 3d) at 0.55 m in core SP. The particularly low Si‐Alk values (Figure S1 in Supporting Information S1) in the coarser sediments below 0.55 m (Piret et al., 2022) may suggest a diminished presence of glacial‐derived ASi and BSi, supporting the hypothesized change in depositional environment post‐GLOF (Piret et al., 2022).

### 3.4 Redox‐Driven Coupling of Si and Fe in Pore Water

Pore water δ^30^Si of the fjord sites (Figure 3d) display strikingly opposing trends to δ^56^Fe (Figure 3b), especially at the sediment Fe(II)‐Fe(III) redox boundary. The highest δ^30^Si (+3.3‰) is observed at the SJ site where the δ^56^Fe minimum is seen (−2.7‰). The SH site shows intermediate δ^30^Si and δ^56^Fe values. Meanwhile, lower δ^30^Si values are observed at the SP and SB sites, where higher δ^56^Fe values are found (Figure 3).

The opposing trends of δ^30^Si and δ^56^Fe between the fjord sites suggests a tight coupling of benthic Si and Fe cycling. This benthic Si‐Fe coupling likely involves DSi adsorption onto freshly precipitated amorphous Fe(III) (oxy)hydroxides (Delstanche et al., 2009) and co‐precipitation of an amorphous Fe(III)‐Si phases (Zheng et al., 2016) that preferentially enriches pore waters with heavier Si isotopes (Geilert, Grasse, Doering, et al., 2020).

Intense pore water advection sustains higher rates of upward Fe^2+^ transport (Section 3.2) and the formation of amorphous Fe(III) minerals in the relatively oxic upper sediment layer. This, in turn, drives additional DSi adsorption and/or co‐precipitation that progressively increases the δ^30^Si of pore waters. The trend of decreasing pore water δ^56^Fe (Figure 3b) and increase of pore water δ^30^Si (Figure 3d) from SB, SP, SH to SJ therefore likely reflects increasing rates of amorphous Fe(III) precipitation driven by pore water advection, and [DSi] adsorption and Si‐Fe co‐precipitation across the fjord sites.

### 3.5 Uncovering the Benthic Coupling of Si and Fe Through Early Diagenetic Modeling

The BRNS‐Si model is used to deconvolve the competing processes in the early diagenetic cycling of Si and to examine the potential significance of benthic Si‐Fe coupling in regulating pore water [DSi] at the fjord sites. Model simulations are performed for the two sites with the highest contrasts in depositional setting and pore water Si isotope composition: (a) SJ—situated proximal to the marine‐terminating glacier, where the largest increase of pore water δ^30^Si is observed (Figure 3d); (b) SB—situated furthest from glaciers, with relatively small pore water δ^30^Si changes (Figure 3d).

**Figure 5:**
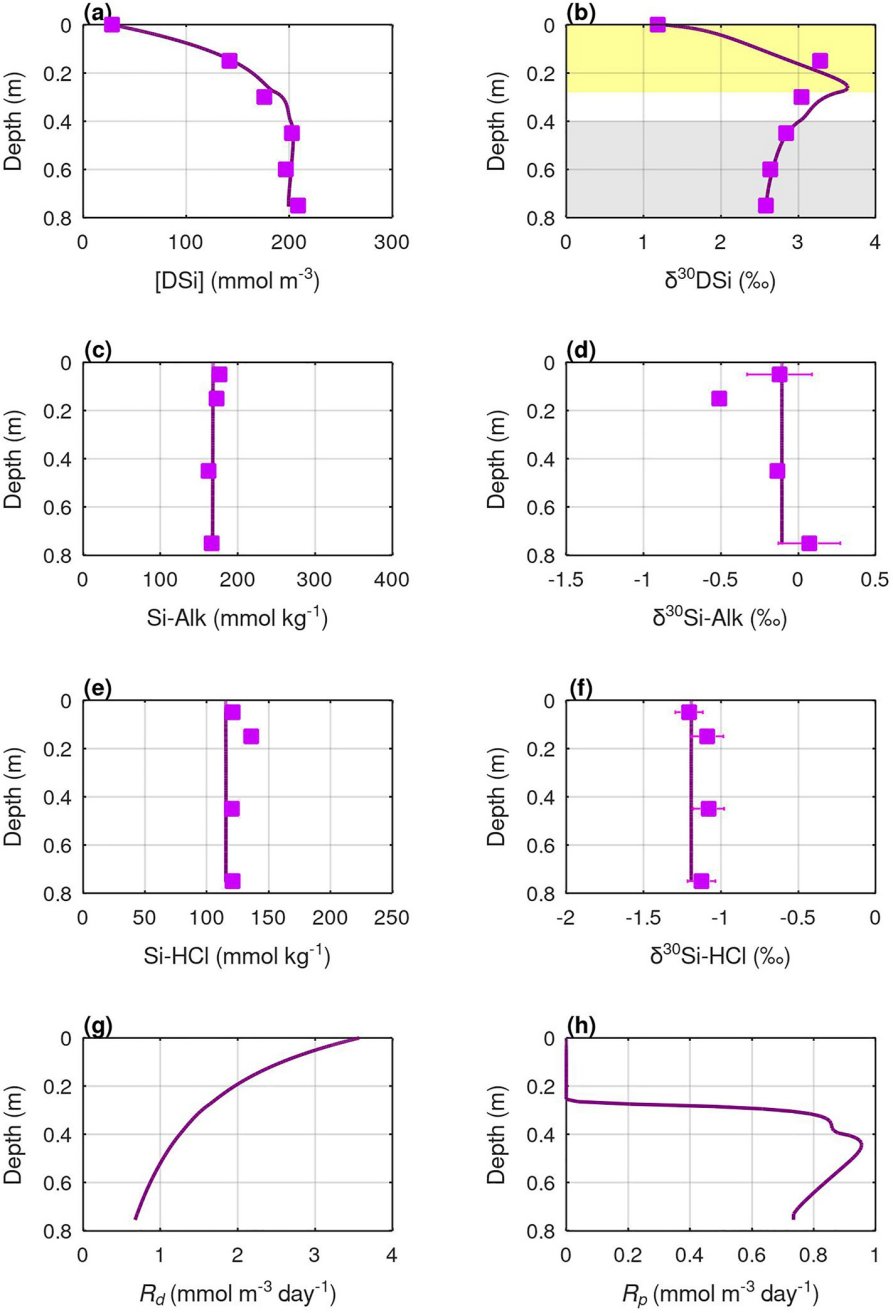
BRNS‐Si model output for site SJ. Pore water DSi (a) concentration, and (b) isotope composition; Sediment Si‐Alk (c) content, and (d) isotope composition; Sediment Si‐HCl (e) content, and (f) isotope composition; Rates of (g) Si‐Alk dissolution, and (h) AuSi precipitation. Lines are model simulations and squares are observational data. Yellow shading marks the depth range of relatively oxic sediments where Si uptake by Fe(III) is enabled in the model. Gray shading indicates the sediment depth range of the ferruginous zone where Si release from Fe dissolution is enabled in the model.

**Figure 6:**
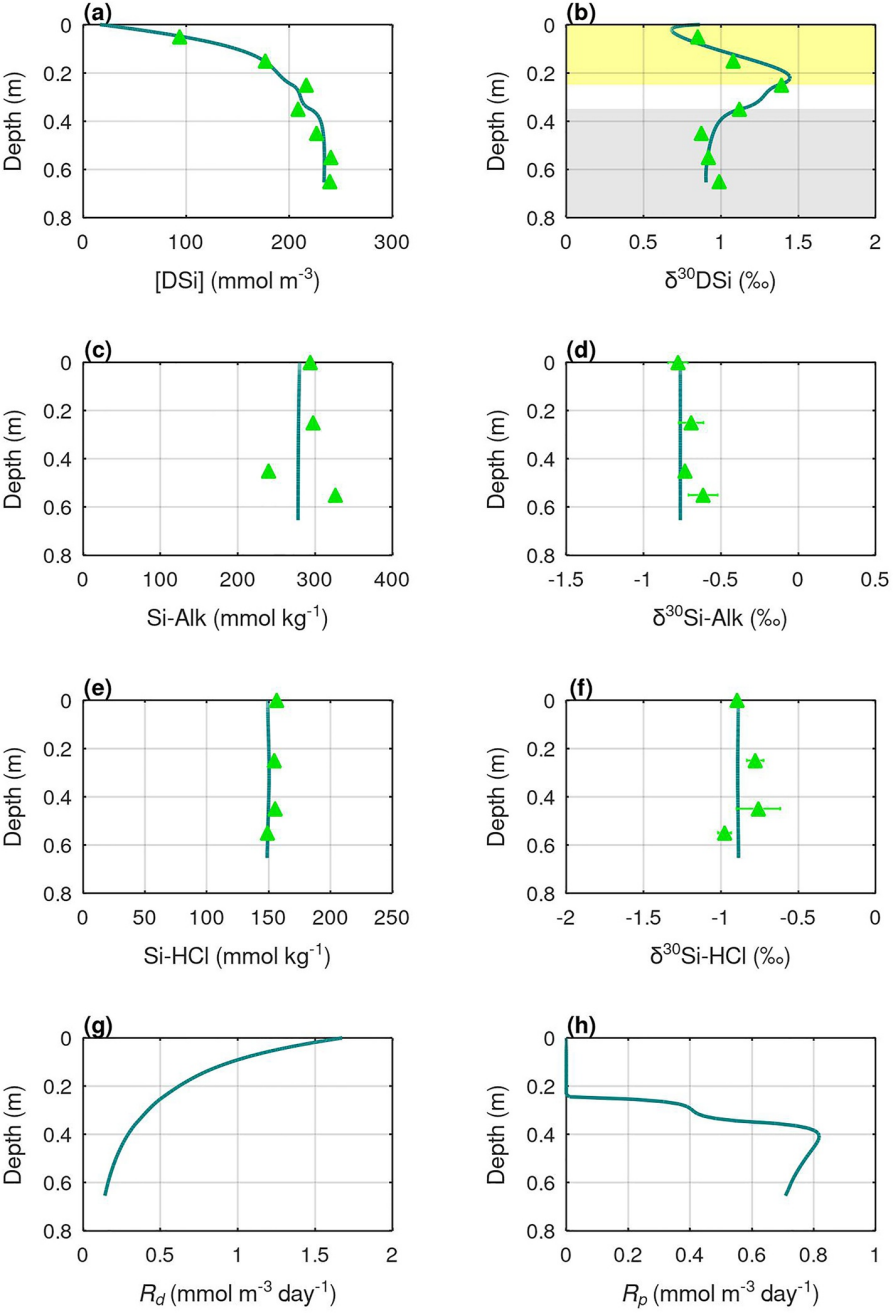
BRNS‐Si model output for site SB. Pore water DSi (a) concentration, and (b) isotope composition; Sediment Si‐Alk (c) content, and (d) isotope composition; Sediment Si‐HCl (e) content, and (f) isotope composition; Rates of (g) Si‐Alk dissolution, and (h) AuSi precipitation. Lines are model simulations and triangles are observational data. Yellow shading marks the depth range of relatively oxic sediments where Si uptake by Fe(III) is enabled in the model. Gray shading indicates the sediment depth range of the ferruginous zone where Si release from Fe dissolution is enabled in the model.

The model's best‐fit of the pore water [DSi] and δ^30^Si profiles (lowest RMSE, Table S3 in Supporting Information S1) can only be achieved with a combination of Si‐Alk dissolution, AuSi precipitation, and redox‐sensitive coupled Si‐Fe reactions (Figures 5 and 6), highlighting the importance of these three diagenetic processes in regulating benthic Si cycling at fjord heads. The modeled dissolution rates of Si‐Alk at SJ are more than twice as high as SB (Figures 5g and 6g), implying that ASi delivered from Jorge Montt (the marine‐terminating glacier) to SJ may be more soluble compared to ASi that have traveled a long distance along the Baker River to reach SB. Model simulations show relatively invariant downcore profiles of sediment Si‐Alk content (Figures 5c and 6c), indicating that only a small fraction of the Si‐Alk pool has dissolved. This finding is consistent with the rapid decline of amorphous silica solubility caused by the incorporation of Al and Fe in the sediments (Aller, 2014; Van Cappellen et al., 2002). Observational data show some downcore variation of sediment Si‐Alk content at SB (Figure 6c) and δ^30^Si‐Alk at SJ (Figure 5d) that are not accounted by the model, which might be due to transient changes in the depositional flux of Si‐Alk and its composition, or other poorly understood diagenetic activities.

The model's best‐fit requires the precipitation of AuSi with saturation concentrations of 180 mmol m^−3^ and 200 mmol m^−3^, and with isotopic fractionations (Δ^30^Si_solid–aqueous_) of −3.0‰ and −2.1‰, at sites SJ and SB respectively (Table S2 in Supporting Information S1). The modeled AuSi saturations are within the range of aluminosilicate saturations found in terrigenous‐rich sediments (Rickert, 2000). Abundant reactive Al and Fe phases in the terrigenous‐rich fjord head sediments (Bianchi et al., 2020) could promote the formation of Fe‐rich AuSi (Geilert, Grasse, Doering, et al., 2020; Isson & Planavsky, 2018) and decrease Si‐Alk solubility (Aller, 2014; Van Cappellen et al., 2002), giving rise to the low asymptotic pore water [DSi] relative to the range of marine observations: 100–800 mmol m^−3^ (Rickert, 2000). The modeled isotopic fractionations associated with AuSi precipitation are at the higher end of the previously published range for aluminosilicate clay mineral formation (−0.5‰–−3.5‰; Frings et al., 2021; Opfergelt & Delmelle, 2012). The higher values could be due to successive cycles of AuSi precipitation and redissolution (Opfergelt & Delmelle, 2012), and/or formation of clay mineral groups that cause greater isotopic fractionations, such as kaolinite (Frings et al., 2021). Depth‐integrated rates of AuSi precipitation at the SJ and SB sites are 0.40 mmol m^−2^ day^−1^ and 0.27 mmol m^−2^ day^−1^, respectively. These rates are within the range previously derived for other delta (7.7 mmol m^−2^ day^−1^; Michalopoulos & Aller, 2004), marginal (0.1–5 mmol m^−2^ day^−1^; Ehlert et al., 2016; Ward et al., 2022a) and tectonically‐active marine settings (0.07–21 mmol m^−2^ day^−1^; Geilert, Grasse, Doering, et al., 2020; Geilert, Grasse, Wallmann, et al., 2020; Luo et al., 2022).

AuSi precipitation mainly occurs after pore water [DSi] reaches the modeled AuSi saturations, which is below 0.25 m sediment depth at the SJ and SB sites (Figures 5 and 6). The increase of pore water δ^30^Si above 0.25 m depth may therefore be attributed mainly to coupled Si‐Fe reactions under oxidizing conditions. Following the argument above, isotopic fractionations (Δ^30^Si_solid–aqueous_) of up to −3.8‰ and −2.2‰ are potentially required for the coupled Si‐Fe reactions at SJ and SB respectively to balance the isotopically light DSi fluid sourced from Si‐Alk dissolution. Such large Si isotopic fractionation may be caused by a combination of DSi adsorption onto Fe(III) (oxy)hydroxides (up to −0.8‰; Delstanche et al., 2009), co‐precipitation of amorphous Fe(III)‐Si phases (up to −3.2‰; Zheng et al., 2016), and successive cycles of DSi‐Fe(III) precipitation and dissolution near a seasonally migrating Fe(II)‐Fe(III) redox boundary (Bianchi et al., 2020; Herbert et al., 2022).

The modeled *R*_up28_ and *R*_up30_ at the SJ site are higher than those at the SB site (Table S2 in Supporting Information S1), consistent with greater DSi adsorption and co‐precipitation with Fe at SJ, as inferred from pore water δ^30^Si observations (Figure 3d; Section 3.4). The modeled data also indicates relatively invariant downcore profiles of sediment Si‐HCl content and δ^30^Si‐HCl, in agreement with observations (Figures 5e, 5f, 6e, and 6f). These modeling results indicate that the addition of Fe‐bound Si to the Si‐HCl pool in the relatively oxic sediment layer and corresponding solubilization in the ferruginous zone result in only subtle variations of the Si‐HCl pool, which could easily be masked by other less reactive Si species that make up the bulk of Si‐HCl phase. These other Si‐HCl species may be Si associated with other less soluble metal oxides (Pickering et al., 2020) or other unidentified secondary weathering products. In addition, the δ^30^Si‐HCl values observed in BMFC sediments show relatively large variation between the study sites (from −0.8‰ to −2.1‰; Figure S1 in Supporting Information S1), and are markedly different from δ^30^Si‐HCl observed in the Gulf of Mexico river plume sediments and the Barents Sea shelf sediments (around −2.9‰; Pickering et al., 2020; Ward et al., 2022b). These observations further highlight the heterogeneous nature of the Si‐HCl phase in the BMFC sediments, which is likely not dominantly composed of Fe (oxy)hydroxide‐bound Si.

### 3.6 Sediment Effluxes of Fe and Si in BMFC

Elevated [dFe] and [DSi] in sediment pore waters relative to core‐top waters (Figure 3) drive diffusive fluxes of the two nutrients out of the sediments along the concentration gradient. Evaluation of the diffusive benthic fluxes of dFe, J_Fe_diffusion_ (mmol m^−2^ day^−1^), requires calculation of the oxidative‐loss of dFe in the oxygenated surface sediment layer, which is incorporated in the equations below (Raiswell & Anderson, 2005; Wehrmann et al., 2014).

(6) $${J\_Fe}_{diffusion}=\frac{\phi_{0}\cdot {\left(D_{m\_Fe}/\theta^{2}\cdot k_{1}\right)}^{0.5}\cdot {\left[dFe\right]}_{p}}{\sinh\left[{\left(\frac{k_{1}\cdot \theta^{2}}{D_{m\_Fe}}\right)}^{0.5}\cdot L_{o}\right]}$$

(7) $$k_{1}=k\left[O_{2}\right]{\left[{OH}^{-}\right]}^{2}$$

*ϕ*_0_ is core‐top (top 1 cm) sediment porosity. *θ* ^2^ is tortuosity, where *θ* ^2^ = 1 − ln(*ϕ*_0_^2^) (Boudreau, 1996). *D*_*m_*Fe_ (m^2^ day^−1^) is the dFe molecular diffusion coefficient in free solution. The thickness of the oxygenated surface sediment layer, *L*_*o*_, is assumed to be around 0.7 cm based on previous oxygen microelectrode measurements (Mulsow et al., 2006). [dFe]_p_ (mmol m^−3^) is the pore water dFe concentration below the oxygenated sediment layer. The first‐order rate constant for Fe^2+^ oxidation, *k*_1_ (day^−1^), is a function of bottom‐water dissolved oxygen concentration, [O_2_] (μmol kg^−1^), constant *k* (μmol^−3^ kg^3^ day^−1^) (Table S5 in Supporting Information S1), and bottom water [OH^−^], which is assumed to be around 0.31 μmol kg^−1^ (approximately equivalent to pH 7.5) in the Patagonian fjords (Mulsow et al., 2006).

Given the asymptotic exponential pore water [DSi] profiles observed at the fjord sites (Figure 3c), diffusive benthic fluxes of DSi, J_Si_diffusion_ (mmol m^−2^ day^−1^), are calculated by Fick's First Law of Diffusion using the following equations (McManus et al., 1995; Ng et al., 2020):

(8) $${J\_Si}_{diffusion}=\phi_{0}\cdot \left(D_{m\_Si}/\theta^{2}\right)\cdot \beta_{DSi}\cdot \left({\left[DSi\right]}_{d}-{\left[DSi\right]}_{0}\right)$$

(9) $${\left[DSi\right]}_{z}={\left[DSi\right]}_{d}-\left({\left[DSi\right]}_{d}-{\left[DSi\right]}_{0}\right)\cdot e^{-{z\beta }_{DSi}}$$

*D*_*m_*Si_ (m^2^ day^−1^) is DSi molecular diffusion coefficient in free solution. *β*_*D*Si_ (m^−1^) is the parameter that describes the curvature of the exponential profiles (Table S6 in Supporting Information S1). [DSi]_0_, [DSi]_d_, and [DSi]_z_ (mmol m^−3^) represent the DSi concentration in core‐top water, asymptotic concentration in pore water, and DSi concentration in pore water at a given sediment depth, *z* (m), respectively.

**Figure 7:**
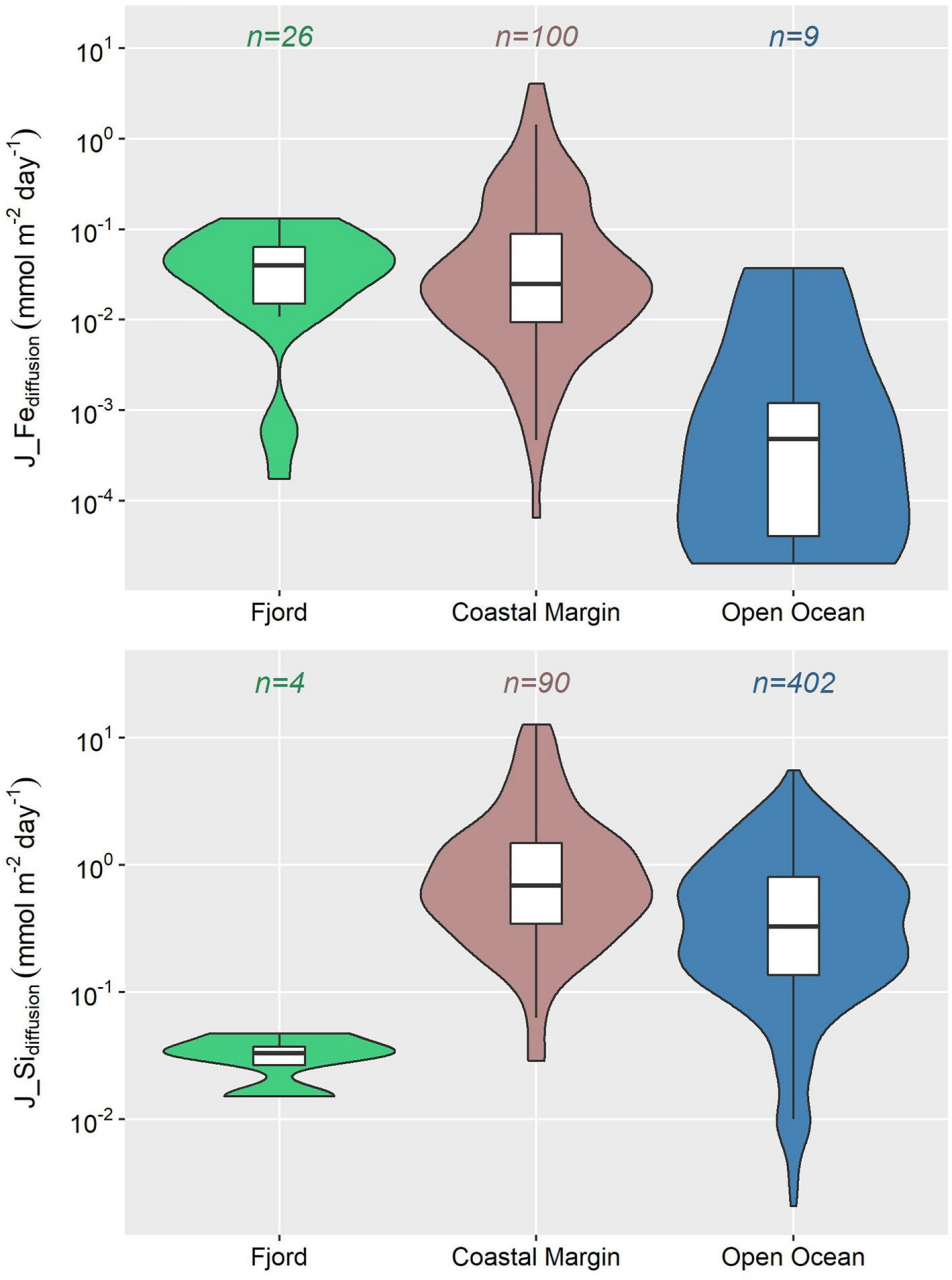
Global data compilation of diffusive benthic Fe and Si fluxes derived from pore water profiles. The data set is presented using logarithmic vertical scales in a combination of box, whisker and violin plots, showing respectively the median and quartiles (Q_1_ or 25th percentile, Q_3_ or 75th percentile), the data spread (1.5 × [Q_3_−Q_1_]), and the data density smoothed by a kernel density estimator. Colored annotations indicate the number of data points available for the three marine settings: fjord, coastal margin, and open ocean. Data are plotted on log scales to handle skewness and to visualize the small values. The plots are created with the R program (R, 2022) using the ggplot2 data visualization package (Wickham, 2016). Thirteen J_Fe_diffusion_ data points (eight from the coastal margin and five from the open ocean) and three J_Si_diffusion_ data points (from the open ocean) have zero values, which are not included in the log scale plots. Our four Patagonian sites represent the first estimations of J_Si_diffusion_ in a fjord setting, while fjordic J_Fe_diffusion_ compiled here also includes estimations from Svalbard fjord sites (Herbert et al., 2021, 2022; Wehrmann et al., 2014). No recalculation has been performed on the published benthic fluxes. Previous pore water Fe and Si studies that have not evaluated benthic fluxes are not included in the compilation. Full references for the compiled data are listed in Table S8 and S9 in Supporting Information S1.

J_Fe_diffusion_ and J_Si_diffusion_ derived for the BMFC sites range from 0.0002 to 0.0243 mmol m^−2^ day^−1^ and 0.015–0.047 mmol m^−2^ day^−1^, respectively (Table 1). Our four Patagonian sites provide the first estimation of the diffusive benthic DSi flux in a fjord setting, as well as the first diffusive benthic dFe flux outside Arctic (Svalbard) fjords. The Patagonian fjords' diffusive benthic dFe fluxes are lower than those from Svalbard fjords (0.01–0.13 mmol m^−2^ day^−1^; Herbert et al., 2021, 2022; Wehrmann et al., 2014). However, diffusive benthic fluxes of dFe in these fjords are larger than the open ocean average and of comparable magnitude to the coastal margin average (Figure 7).

**Table 1:** Diffusion Fluxes of Fe and Si Across the Sediment‐Water Interface Calculated for the Fjord Sites

Site	J_Fe_diffusion_ (mmol m^−2^ day^−1^)	J_Si_diffusion_ (mmol m^−2^ day^−1^)
SJ	0.0005 ± 0.0003	0.015 ± 0.004
SH	0.0007 ± 0.0003	0.047 ± 0.007
SP	0.0002 ± 0.0001	0.035 ± 0.012
SB	0.0243 ± 0.0118	0.032 ± 0.007

In contrast, diffusive benthic DSi fluxes at the fjord sites are small relative to other marine settings (Figure 7). Given the knowledge of sediment Si‐HCl and Si‐Alk content (mmol kg^−1^), sediment bulk density, *ρ*_*b*_ (kg m^−3^), and sedimentation rates, *S* (m day^−1^) (Table S7 in Supporting Information S1), the sediment burial rates of the reactive silica phases, J_RSi_burial_ at the fjord sites are estimated to range from 14 to 264 mmol m^−2^ day^−1^ (Equation 10).

(10) $$J\_{RSi}_{burial}=\left(\left[Si-HCl\right]+\left[Si-Alk\right]\right)\cdot \rho_{b}\cdot S$$

These values suggest that diffusive benthic DSi fluxes are only returning 0.006%–0.25% of the sediment's reactive Si back to the water column, which is very small compared to the global marine average J_Si_diffusion_: J_RSi_burial_ (>500%) (Tréguer et al., 2021). The small J_Si_diffusion_: J_RSi_burial_ (0.006%–0.25%) of the study area is consistent with the high sedimentation rates at the fjord sites (>0.7 cm yr^−1^, Table S1 in Supporting Information S1) compared to the ocean (Bianchi et al., 2020), which supports reactive Si burial in these fjord sediments.

### 3.7 The Role of the Benthic Flux in Fjord Nutrient Cycling

Our findings support active benthic cycling that sustains a significant flux of dFe, but a smaller than expected flux of DSi from the sediments at the fjord heads to the overlying fjord waters. These results have important implications for the ongoing debate on the glacier‐to‐ocean transport of the two nutrients, and for the modulation of nutrient ratios and phytoplankton composition in fjords and the adjacent coastal ocean (Hopwood et al., 2020). The elevated sediment dFe efflux relative to the open ocean average (Figure 7), despite high sedimentation rates, highlights the importance of benthic cycling in processing glacially‐sourced solid Fe phases to dissolved forms through intense redox cycling. These findings support the notion that fjord sediments recycle significant fluxes of potentially‐bioavailable Fe to the overlying water column (Herbert et al., 2021; Laufer‐Meiser et al., 2021; Wehrmann et al., 2014), which could be supplied to downstream ecosystems via a combination of upwelling, estuarine circulation and fjord‐shelf exchange (Bianchi et al., 2020; Hopwood et al., 2020).

A low sediment DSi efflux at the fjord sites relative to other marine settings (Figure 7) is likely the combined result of high sedimentation rates at the fjord heads, low pore water [DSi] asymptotes, and the coupling of benthic Si‐Fe cycling. Indeed, when Si adsorption and co‐precipitation with Fe (and co‐dissolution) are “turned off” in the BRNS‐Si model (i.e., *R*_up28_, *R*_up30_, *R*_re28_, *R*_re30_ are zero), the diffusive benthic DSi fluxes calculated from the new pore water [DSi] profiles (Figure S2 in Supporting Information S1) are 0.043 mmol m^−2^ day^−1^ for SJ and 0.080 mmol m^−2^ day^−1^ for SB, which are 2–3 times larger than the original observations (Table 1). The experiment above supports the role of coupled Si‐Fe reactions in suppressing the diffusive benthic DSi flux. This coupling is thought to be particularly pronounced at fjord heads, where there are heightened supplies of reactive Fe from the melting glaciers (Henkel et al., 2018; Pryer, Hawkings, et al., 2020; Wehrmann et al., 2014) and oxidative precipitation of amorphous Fe(III) minerals in the upper sediment layer. Whilst there is a ready supply of dissolvable ASi from the melting glaciers (Pryer, Hawkings, et al., 2020), efficient burial of this phase deposited beneath the fjord heads and the low DSi return flux out of these sediments may limit the amount of bioavailable Si that gets transported to downstream ecosystems. While this study focuses on sites at the Patagonian fjord heads, benthic Si and Fe cycling at sites further away from glacier calving front and riverine outflows may exhibit different trends, given the lower sedimentation rates, changes in primary productivity, organic matter supply and sedimentary Fe(III) reduction rates down the fjords (Herbert et al., 2021; Laufer‐Meiser et al., 2021). Further constraining the evolution of benthic Si‐Fe coupling along the fjords requires future dedicated sampling campaigns that will obtain sediment cores and pore water samples from middle and outer fjords, in addition to fjord heads.

The sediment nutrient effluxes derived here are likely conservative estimates, as the benthic flux is not solely driven by diffusion. Other processes that contribute to benthic nutrient fluxes at fjords include benthic macrofaunal activities such as bioturbation and bio‐irrigation, pore water release driven by bottom currents and physical disturbance such as iceberg scouring (Schulz & Zabel, 2006; Wehrmann et al., 2014). These processes will likely result in a net sediment efflux that is higher than the flux maintained by diffusion alone (Ng et al., 2020; Wehrmann et al., 2014). Our study provides important insights into the primary geochemical processes that govern the return fluxes of nutrient Si and Fe deposited in the sediments back to fjord waters. These benthic geochemical processes should be considered when evaluating nutrient cycling in fjords and the future evolution of nutrient delivery to downstream coastal areas amid rapidly retreating glacier cover.

## 4 Conclusions

Analyses of pore water and sequential sediment chemical extractions were performed on four sediment cores acquired from the head of the BMFC in Chilean Patagonia. The fjord sediments exhibit some of the highest published concentrations of dFe in pore waters (up to 800 mmol m^−3^), which are associated with an elevated supply of highly reactive Fe (oxy)hydroxides and other Fe‐rich reactive minerals from upstream glaciers. Delivery of Fe as colloids/nanoparticulate aggregates may also play a role in influencing the isotopic composition of Fe in pore waters and its transport away from sediments, as has recently been observed in open ocean sediments.

Our stable isotope analyses and model simulation suggest that pore water DSi at the fjord sites are governed by the dissolution of glacially‐derived ASi and BSi, authigenic precipitation of clays, and a close coupling between the benthic cycling of Si and Fe. Fjord sites that show lower pore water δ^56^Fe, indicative of more intense Fe redox cycling and in‐situ production of Fe (oxy)hydroxides, is concurrent with elevated pore water δ^30^Si. This coupling of Si and Fe isotopes can be explained by Si adsorption onto the newly produced Fe (oxy)hydroxides from upwardly diffusing Fe^2+^ rich pore water and co‐precipitation of amorphous Fe‐Si minerals.

The tight coupling of benthic Si and Fe cycling has an impact on the diffusive fluxes of the two nutrients across the sediment‐water interface. Sediment effluxes of dFe at the Patagonian fjord sites, maintained by the high pore water concentrations, are relatively large (up to 0.02 mmol m^−2^ day^−1^) compared to open ocean sites (typically <0.001 mmol m^−2^ day^−1^). In contrast, sediment effluxes of DSi in fjord sediments (0.02–0.05 mmol m^−2^ day^−1^) are relatively low (typical ocean values are >0.1 mmol m^−2^ day^−1^). We hypothesize that upwardly diffusing Si is trapped in the solid phase through adsorption and co‐precipitation with Fe oxyhydroxides, and there is efficient burial of dissolvable reactive Si phases due to very high sedimentation rates. At present, benthic cycling at Fe‐rich fjord heads sustains a significant release of dFe but restricts the supply of DSi that can be transported to downstream ecosystems.

## Conflict of Interest

The authors declare no conflicts of interest relevant to this study.

## Supporting information

Supporting Information S1

Data Set S1

## Data Availability

The pore water, sediment Si‐Alk, Si‐HCl data reported in this paper are listed in the Supporting Information S1. These measured data, as well as the BRNS‐Si model outputs are also available in the UK Polar Data Centre under Open Government Licence V3.0 at the following link: https://doi.org/10.5285/5AF9E5CF-657D-4640-8FF3-DD3EC2F43367 (Ng et al., 2022). BRNS‐Si model codes used in this study are available under GNU General Public License Version 3 at the following link: https://github.com/CoderHCN/Patagonian-fjord-silicon-model-BRNS-code-and-output. The core equations and input parameters for the BRNS‐Si model were generated and compiled with the Maple (2016) software (Maple, 2016).
