# Supplementary material for: Benthic Dissolved Silicon and Iron Cycling at Glaciated Patagonian Fjord Heads
Source: Global Biogeochem Cycles. 2022 Nov 25;36(11):e2022GB007493. doi: 10.1029/2022GB007493 (PMC9786927; doi:10.1029/2022GB007493)
Supplement: Supplementary file 1 — Supporting Information S1 [file GBC-36-e2022GB007493-s002.pdf]

Supporting Information for

Benthic dissolved silicon and iron cycling at glaciated Patagonian fjord heads

Hong Chin Ng[1,2,*], Jon R. Hawkings[3], Sebastien Bertrand[4], Brent A. Summers[5], Matthias Sieber[5], Tim M. Conway[5], Felipe S. Freitas[1,6], James P.J. Ward[1], Helena V. Pryer[7,8], Jemma L. Wadham[7,9], Sandra Arndt[6], Katharine R. Hendry[1,10]

[1] School of Earth Sciences, University of Bristol, UK

[2] Ifremer, Université Bretagne Occidentale, CNRS, Geo-Ocean, 29280, Plouzané, France

[3] Department of Earth and Environmental Science, University of Pennsylvania, Philadelphia, USA

[4] Renard Centre of Marine Geology, Ghent University, Gent, Belgium

[5] College of Marine Science, University of South Florida, St Petersburg, FL, USA

[6] BGeosys, Department of Geosciences, Université libre de Bruxelles, Brussels, CP160/03 1050, Belgium.

[7] Bristol Glaciology Centre, School of Geographical Sciences, University of Bristol, UK

[8] Department of Earth Sciences, University of Cambridge, UK

[9] Centre for Arctic Gas Hydrate, Environment and Climate (CAGE), Department of Geosciences, UiT The Arctic University of Norway, N-9037 Tromsø, Norway.

[10] Polar Oceans Team, British Antarctic Survey, Cambridge, UK.

* Corresponding author: hn9381@bristol.ac.uk ; hcng@ifremer.fr

Content:

Equation S1–S12.	Silicon isotope equations used in BRNS-Si model.
Equation S13–S14.	RMSE equations.
Figure S1.	Sediment reactive silica phases at the fjord sites.
Figure S2.	Model test: no benthic silicon-iron coupling scenario.
Table S1.	Brief description of sediment cores.
Table S2.	BRNS-Si model parameters and boundary conditions.
Table S3.	Goodness of fit measures between observational and simulated data.
Table S4.	Site glacial cover and reactive iron supply.
Table S5.	Parameters used to calculate diffusive benthic flux of iron.
Table S6.	Parameters used to calculate diffusive benthic flux of silicon.
Table S7.	Sediment burial fluxes of reactive Si phases.
Table S8.	Global compilation of diffusive benthic dissolved iron flux data reference list.
Table S9.	Global compilation of diffusive benthic dissolved silicon flux data reference list.

Silicon isotope equations used in BRNS-Si model

The BRNS-Si model simulates Si isotope composition with explicit ^{28}Si and ^{30}Si isotope concentrations, which can be converted from instrumental measurements of δ^{30}Si using Equation S1–S4 (Cassarino et al., 2020; Ward et al., 2022b):

$$\left(\frac{^{30}\mathrm{Si}}{^{28}\mathrm{Si}}\right) = \left(1 + \delta^{30}\mathrm{Si}_{\mathrm{measured}} \cdot 1000\right) \times \left(\frac{^{30}\mathrm{Si}}{^{28}\mathrm{Si}}\right)_{\mathrm{reference}} \quad \text{(S1)}$$

$$\left(\frac{^{29}\mathrm{Si}}{^{28}\mathrm{Si}}\right) = \left(1 + \delta^{29}\mathrm{Si}_{\mathrm{measured}} \cdot 1000\right) \times \left(\frac{^{29}\mathrm{Si}}{^{28}\mathrm{Si}}\right)_{\mathrm{reference}} \quad \text{(S2)}$$

$$\left[^{28}\mathrm{Si}\right] = \frac{1}{1+\frac{^{29}\mathrm{Si}}{^{28}\mathrm{Si}}+\frac{^{30}\mathrm{Si}}{^{28}\mathrm{Si}}} \quad \text{(S3)}$$

$$\left[^{30}\mathrm{Si}\right] = \frac{\frac{^{30}\mathrm{Si}}{^{28}\mathrm{Si}}}{1+\frac{^{29}\mathrm{Si}}{^{28}\mathrm{Si}}+\frac{^{30}\mathrm{Si}}{^{28}\mathrm{Si}}} \quad \text{(S4)}$$

$(^{30}\mathrm{Si}/^{28}\mathrm{Si})_{\mathrm{reference}}$ and $(^{29}\mathrm{Si}/^{28}\mathrm{Si})_{\mathrm{reference}}$ are equal to 0.0341465 and 0.0507446 respectively (Ding et al., 2005).

Potential isotopic fractionation of Si associated with the dissolution of Si-Alk and the precipitation of AuSi are simulated by the BRNS-Si model with Equation S5–S6 and Equation S7–S8, respectively (Cassarino et al., 2020; Ward et al., 2022b):

$$R_{d28} = \left(\frac{\propto_d \cdot [^{28}\text{Si-Alk}]}{[^{30}\text{Si-Alk}] + \propto_d \cdot [^{28}\text{Si-Alk}]}\right) \cdot R_d \quad \text{(S5)}$$

$$R_{d30} = \left(\frac{[^{30}\text{Si-Alk}]}{[^{30}\text{Si-Alk}] + \propto_d \cdot [^{28}\text{Si-Alk}]}\right) \cdot R_d \quad \text{(S6)}$$

$$R_{p28} = \left(\frac{\propto_p \cdot [^{28}\mathrm{DSi}]}{[^{30}\mathrm{DSi}] + \propto_p \cdot [^{28}\mathrm{DSi}]}\right) \cdot R_p \quad \text{(S7)}$$

$$R_{p30} = \left(\frac{[^{30}\mathrm{DSi}]}{[^{30}\mathrm{DSi}] + \propto_p \cdot [^{28}\mathrm{DSi}]}\right) \cdot R_p \quad \text{(S8)}$$

R_{d28} and R_{d30} are kinetic dissolution rates (R_d, Equation 2 in main text) of the light and heavy Si-Alk isotopes respectively. R_{p28} and R_{p30} are kinetic precipitation rates (R_p, Equation 3 in main text) of the light and heavy DSi isotopes respectively. α_d and α_p are isotopic fractionation factors of the dissolution and precipitation reactions respectively, with value of 1.000 indicating no isotopic fractionation.

Potential changes in Si-HCl isotope composition following the rates of uptake of pore water ^{28}Si and ^{30}Si by Fe (oxy)hydroxides (R_{up28}, R_{up30}), and the rates of release of ^{28}Si and ^{30}Si into pore water due to reductive dissolution of Fe (oxy)hydroxides (R_{re28}, R_{re30}), are simulated by the BRNS-Si model with Equation S9–S12:

$$\frac{\partial \sigma^{28}Si\text{-}HCl}{\partial t} = \cdots + R_{up28} \quad \text{if } z_{ox\text{-}up} < z < z_{ox\text{-}low} \tag{S9}$$

$$\frac{\partial \sigma^{30}Si\text{-}HCl}{\partial t} = \cdots + R_{up30} \quad \text{if } z_{ox\text{-}up} < z < z_{ox\text{-}low} \tag{S10}$$

$$\frac{\partial \sigma^{28}Si\text{-}HCl}{\partial t} = \cdots - R_{re28} \quad \text{if } z_{fe\text{-}up} < z < z_{fe\text{-}low} \tag{S11}$$

$$\frac{\partial \sigma^{30}Si\text{-}HCl}{\partial t} = \cdots - R_{re30} \quad \text{if } z_{fe\text{-}up} < z < z_{fe\text{-}low} \tag{S12}$$

… is $\frac{\partial}{\partial z}\left[(D_{bio})\frac{\partial \sigma C_{Si}}{\partial z}\right] - \frac{\partial \sigma \omega C_{Si}}{\partial z}$ from Equation 1 in the main text, note that $D_{m\_Si} = 0$ and $\alpha_{irr} = 0$ for solid phases. $z_{ox\text{-}up}$ and $z_{ox\text{-}low}$ are the upper and lower depth limits of the oxidizing layer, and $z_{fe\text{-}up}$ and $z_{fe\text{-}low}$ are the upper and lower depth limits of the ferruginous zone (Table S2).

Root-mean-square-error equations

$$\text{RMSE} = \sqrt{\frac{1}{n}\sum_{i=1}^{n}(x_i - \hat{x}_i)^2} \tag{S13}$$

$$\text{NRMSE} = \frac{\text{RMSE}}{x_{max} - x_{min}} \tag{S14}$$

i represents the variable of interest. *n* is the number of data. x_i, x_{max} and x_{min} are the observation, observation's maximum and minimum values respectively. $\hat{x}_i$ is the model simulation.

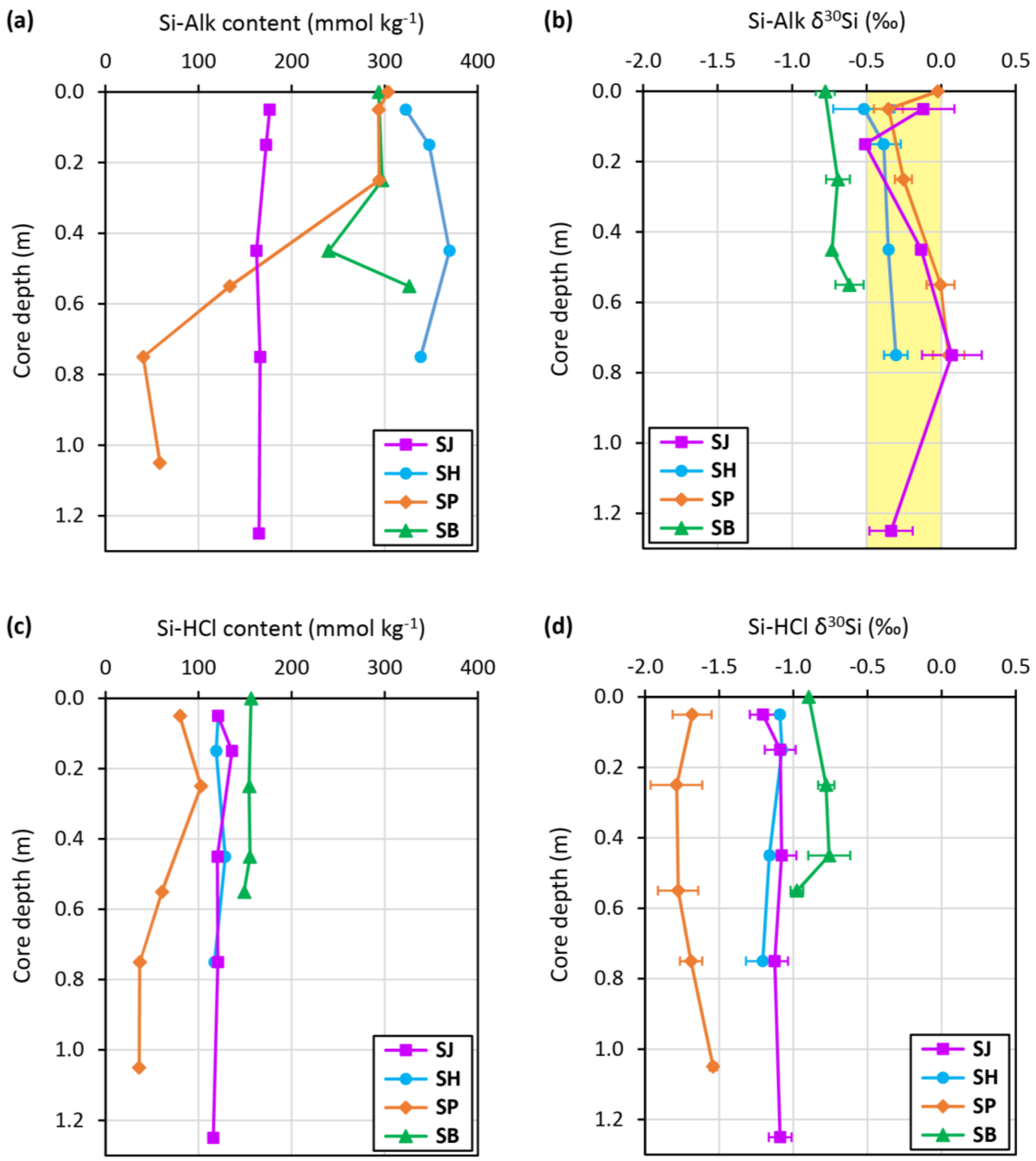

Figure S1. Analytical results of sediment reactive silica phases at the four fjord sites. Sediment depth profiles of **(a)** Si-Alk content, **(b)** Si-Alk δ^{30}Si, **(c)** Si-HCl content, and **(d)** Si-HCl δ^{30}Si data. Error bars in (b) and (d) represent 95% confidence intervals of δ^{30}Si measurements; some error bars are smaller than the symbols. The yellow box indicates the range of glacial ASi δ^{30}Si endmembers (Hatton et al., 2019; Hawkings et al., 2018).

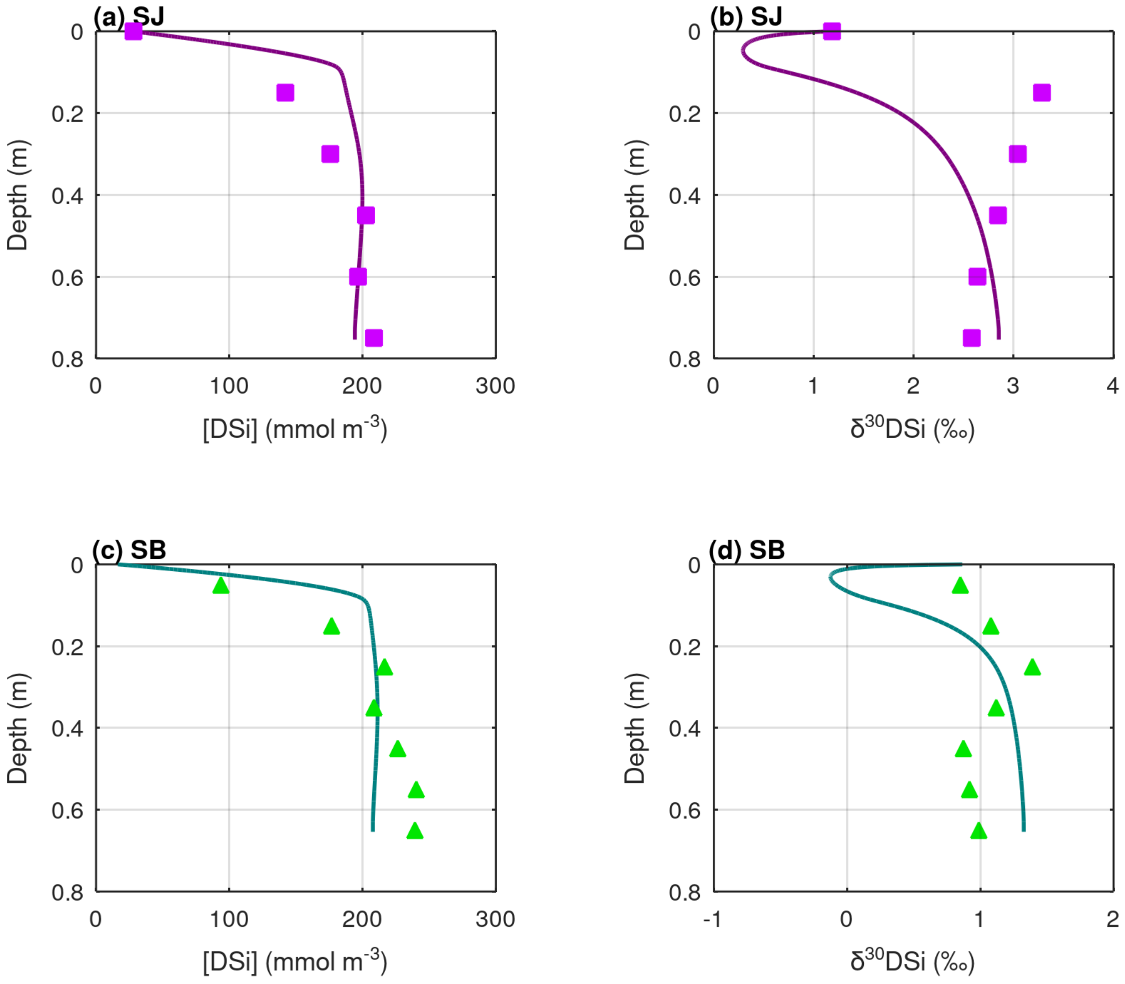

Figure S2. BRNS-Si model experiment testing the scenario of no Si-Fe coupled reactions in fjord sediments. In this scenario, zero values have been assigned to model parameters (R_{up28}, R_{up30}, R_{re28}, R_{re30}) defining the uptake of Si by Fe(III) in the relatively oxic sediment layer and the release of Si from Fe reduction in the ferruginous zone. Pore water DSi **(a)** concentration and **(b)** isotope composition for the SJ site; and pore water DSi **(a)** concentration and **(b)** isotope composition for the SB site. Lines are model simulations, while squares and triangles are observational data.

Table S1. Brief information of the sediment cores: location, lithology, overall porosity and sedimentation rates.

Core site	Corresponding core label	Latitude (° S)	Longitude (° W)	Water depth (m)	Core depth interval (m)	Lithology	Average porosity	Sedimentation rate (cm yr^{-1})	Reference
SJ	PI17-FC-S19	48.228	73.502	106	0 – 1.0	Mainly composed of mud, with notable amount (up to 10%) of coarse (>63 µm) ice-rafted sand occurring periodically at around every 0.2 m interval.	0.63	20	Boldt (2014); De Wilde (2016)
SH	FC17-14	47.679	73.715	203	0 – 0.75	Dominantly mud, with laminae of coarser silt and fine sand.	0.58	2.82	Piret et al. (2021)
SP	PI17-FC-S10	48.179	73.347	248	0 – 0.5	Dominantly mud, with occasional silty laminae.	0.58	0.75	Piret et al. (2022)
					0.5 – 1.0	Mud with frequent silty-sandy laminae deposits.	0.28		
SB	FC17-10	47.787	73.61	151	0 – 0.65	Primarily composed of silt (A), with relatively frequent occurrence of finer silt layers (B).	0.51	3.26	Vandekerkhove et al. (2021)

Table S2. BRNS-Si model parameters and boundary conditions. Values are categorized as 'Independent' (I) for direct laboratory analyses of the fjord samples or field measurements at the fjord sites; 'Constrained' (C) for those based on evaluation from other global sites; and 'Model-derived' (M) for those determined from the simulation best fit with the observed fjords' sediment profiles.

Parameter	Unit specified in model	Type	SJ Site	SB Site	Notes/Reference
Bottom depth of simulation, L_M	cm	-	75	65	-
Run time	year	-	7.5	42.6	Steady-state conditions are reached relatively quickly – within 3.75 and 12.75 years of simulation for SJ and SB respectively, supporting the use of BRNS for sites with high sedimentation rates.
Temperature, T	^{o}C	I	9.0	8.4	-
Salinity, S	PSU	I	33.50	33.80	-
Core-top porosity, ϕ_0	-	I	0.56	0.64	Exponential best-fits of the porosity profiles were computed with the equations: $\phi_z = \phi_x + (\phi_0 - \phi_x).(1 - \exp(-z.\beta_\phi))$ for the SJ site, and $\phi_z = \phi_x + (\phi_0 - \phi_x).\exp(-z.\beta_\phi)$ for the SB site, where z is sediment depth.
Bottom porosity, ϕ_x	-	I	0.67	0.50	
Porosity coefficient, β_ϕ	-	M	0.0273	0.1142	
[1] Bioturbation coefficient, D_{bio}	cm^2 yr^{-1}	C	0.055	0.055	(Oleszczuk et al., 2019)
[1] Bioturbation depth, z_{bio}	cm	C	10	10	
[1] Bioirrigation coefficient, α_0	yr^{-1}	C	0.405	0.405	
[1] Bioirrigation attenuation depth, x_{irr}	cm	C	3.5	3.5	(Freitas et al., 2020; Ward et al., 2022b); Bioirrigation rate, $\alpha_{irr} = \alpha_0 . \exp(-z/x_{irr})$
Sedimentation rate, ω	cm yr^{-1}	I	20	3.05	De Wilde (2016); Vandekerkhove et al. (2021)
Molecular diffusion coefficient of Si, $D_{m\text{-}Si}$	cm^2 yr^{-1}	C	201	196	Sediment diffusion coefficient, $D_{sed\text{-}Si} = D_{m\text{-}Si} / (1\text{-}\ln(\phi^2))$; ϕ is porosity (Boudreau, 1996).
[^{28}DSi]	mol cm^{-3}	I	2.584 x 10^{-8}	1.583 x 10^{-8}	Core-top [DSi] for SB was extrapolated based on least squares exponential fitting of available pore water data (Fig. 3 in main text).
[^{30}DSi]	mol cm^{-3}	I	8.835 x 10^{-10}	5.412 x 10^{-10}	
[^{28}Si-Alk]	mol cm^{-3}	I	4.067 x 10^{-4}	6.770 x 10^{-4}	-
[^{30}Si-Alk]	mol cm^{-3}	I	1.388 x 10^{-5}	2.310 x 10^{-5}	-
[^{28}Si-HCl]	mol cm^{-3}	I	2.006 x 10^{-4}	2.596 x 10^{-4}	-
[^{30}Si-HCl]	mol cm^{-3}	I	6.843 x 10^{-6}	8.858 x 10^{-6}	-
Dissolution rate constant, k_{diss}	yr^{-1}	M	3.0 x 10^{-3}	0.85 x 10^{-3}	-
Dissolution coefficient *ad*	-	M	0.022	0.04	-
Dissolution coefficient *bd*	-	M	0.07	0.05	-
Amorphous silica saturation, [ASi]$_{sat}$	mol cm^{-3}	C	8.0 x 10^{-7}	8.0 x 10^{-7}	Loucaides et al. (2012); Willey (1974)
Isotopic fractionation factor of Si-Alk dissolution, α_d	-	C	1.000	1.000	Wetzel et al. (2014)
Precipitation rate constant, k_{precip}	yr^{-1}	M	5.0 x 10^{-5}	2.5 x 10^{-5}	-
Precipitation coefficient *ap*	-	M	0.13	0.10	-

Precipitation coefficient *bp*	-	M	0.05	0.06	-
Authigenic silicate saturation, $[AuSi]_{sat}$	mol cm^{-3}	M, C	1.8 x 10^{-7}	2.0 x 10^{-7}	Khalil et al. (2007); Rickert (2000)
Isotopic fractionation factor of AuSi precipitation, α_p	-	M, C	1.003	1.0021	Opfergelt and Delmelle (2012)
Uptake of ^{28}Si by Fe, R_{up28}	mol cm^{-3} yr^{-1}	M	3.3 x 10^{-7}	2.0 x 10^{-7}	-
Uptake of ^{30}Si by Fe, R_{up30}	mol cm^{-3} yr^{-1}	M	11.2471 x 10^{-9}	6.82018 x 10^{-9}	-
Upper depth limit of oxidizing layer, z_{ox-up}	cm	I	0	0	-
Lower depth limit of oxidizing layer, z_{ox-low}	cm	I	28	25	-
Release of ^{28}Si from Fe, R_{re28}	mol cm^{-3} yr^{-1}	M	0.7 x 10^{-7}	2.0 x 10^{-7}	-
Release of ^{30}Si from Fe, R_{re30}	mol cm^{-3} yr^{-1}	M	2.38575 x 10^{-9}	6.82018 x 10^{-9}	-
Upper depth limit of ferruginous zone, z_{fe-up}	cm	I	40	35	-
Lower depth limit of ferruginous zone, z_{fe-low}	cm	I	75	65	-

[1] Despite the complex and heterogeneous nature of bioturbation and bio-irrigation in the sediments, simplified representations were used given the model's capacity.

Table S3. Goodness of fit measures between pore water DSi observation and simulated data. RMSE and NRMSE are calculated based on Equation S13–S14.

Site	[DSi]			δ^{30}Si		
	RMSE	NRMSE	r^2	RMSE	NRMSE	r^2
SJ	22.1 µM	0.12	0.97	0.41‰	0.20	0.75
SB	8.0 µM	0.05	0.97	0.08‰	0.15	0.85

Table S4. Glacial cover and yield of FeA (ascorbate-extractable Fe: mainly composed of freshly precipitated, easily reducible ferrihydrites) upstream of the four fjord sites. Data of SH, SP and SB are from Pryer et al. (2020).

Site	Upstream glacial cover (%)	Upstream yield of FeA (g m^{-2} day^{-1})
SJ	100	Expected to be very high (Laufer-Meiser et al., 2021)
SH	71.0	4.5×10^{-3}
SP	18.5	0.4×10^{-3}
SB	7.6	0.5×10^{-3}

Table S5. Parameters involved in the calculation of diffusion fluxes of Fe across the sediment-water interface for the four fjord sites (Equation 6 & 7).

Site	ϕ_0	θ^2	D_{m_Fe} (m^2 day^{-1}) [i]	$[dFe]_p$ (mmol m^{-3}) [ii]	$[O_2]$ (µmol kg^{-1})	k (µmol^{-3} kg^3 day^{-1}) [iii]	k_1 (day^{-1})
SJ	0.53 ± 0.04	2.25 ± 0.14	$(3.80 \pm 0.01) \times 10^{-5}$	3.04 ± 0.35	173.4 ± 0.1	0.350 ± 0.009	5.8 ± 1.5
SH	0.77 ± 0.02	1.53 ± 0.05	$(3.70 \pm 0.01) \times 10^{-5}$	1.01 ± 0.16	158.5 ± 0.1	0.338 ± 0.009	5.1 ± 1.3
SP	0.67 ± 0.04	1.81 ± 0.11	$(3.68 \pm 0.01) \times 10^{-5}$	0.41 ± 0.05	156.4 ± 0.1	0.335 ± 0.009	5.0 ± 1.3
SB	0.62 ± 0.03	1.95 ± 0.09	$(3.72 \pm 0.01) \times 10^{-5}$	72.91 ± 8.35	155.0 ± 0.1	0.341 ± 0.009	5.0 ± 1.3

[i] D_{m_Fe} was determined based on a previous evaluation of D_{m_Fe} values at a range of temperature (Schulz & Zabel, 2006), and bottom water temperatures proximal to the core sites.
[ii] $[dFe]_p$ was estimated pore water dFe concentration beneath the inferred oxygen penetration depth (0.7 cm) based on linear interpolation of the closest [dFe] measurements.
[iii] k was evaluated based on an empirical relationship: $\log k = 21.56 - 1545/T - 3.29I^{0.5} + 1.52I$ (Millero et al., 1987), where T is temperature in kelvin, and I is the ionic strength, which is assumed to be 0.723 for the bottom seawater at the fjord sites, given measured salinity of about 34 PSU.

Table S6. Parameters involved in the calculation of diffusion fluxes of Si across the sediment-water interface for the four fjord sites (Equation 8 & 9).

Site	D_{m_Si} (m^2 day^{-1}) [i]	$[DSi]_0$ (mmol m^{-3})	$[DSi]_d$ (mmol m^{-3}) [ii]	β_{DSi} (m^{-1}) [ii]
SJ	$(5.5 \pm 0.1) \times 10^{-5}$	28.0 ± 0.7	206 ± 5	6.5 ± 0.6
SH	$(5.3 \pm 0.1) \times 10^{-5}$	21.4 ± 0.5	234 ± 3	8.3 ± 0.4
SP	$(5.3 \pm 0.1) \times 10^{-5}$	25.4 ± 0.6	261 ± 9	7.6 ± 1.3
SB	$(5.4 \pm 0.1) \times 10^{-5}$	17.2 ± 0.4	235 ± 6	8.6 ± 0.8

[i] D_{m_Si} was determined based on a previous experimental study that evaluated D_{m_Si} values at a range of temperature (Rebreanu et al., 2008), and bottom water temperatures proximal to the core sites.
[ii] $[DSi]_d$ and β_{DSi} were derived from Equation (7) through least squares exponential fit of the pore water [DSi] profiles (Fig. 3a), using the Regression Wizard function in SigmaPlot (Systat Software Inc.).

Table S7. Sediment burial fluxes of reactive Si phases calculated for the four fjord sites and the parameters involved in the calculation (Equation 10).

Site	Si-HCl (mmol kg^{-1}) [i]	Si-Alk (mmol kg^{-1}) [i]	ρ_b (kg m^{-3})	S (m day^{-1})	J_RSi_{burial} (mmol m^{-2} day^{-1})
SJ	123 ± 18	169 ± 42	1660 ± 210	$(55 \pm 14) \times 10^{-5}$	264 ± 155
SH	121 ± 18	345 ± 86	1750 ± 210	$(7.7 \pm 0.5) \times 10^{-5}$	63 ± 26
SP [ii]	91 ± 14	297 ± 74	1730 ± 130	$(2.1 \pm 0.8) \times 10^{-5}$	14 ± 9
SB	154 ± 23	289 ± 72	1860 ± 150	$(8.9 \pm 1.9) \times 10^{-5}$	74 ± 38

[i] Averages of measured sediment core Si-HCl and Si-Alk content (Fig. S1).
[ii] Given evidence for a change in depositional environment that is not representative of the current fjord environment below ~0.5 m depth of core SP (Section 3.1.1) (Piret et al., 2022), only data from the top 0.5 m depth were used for J_RSi_{burial} calculation.

Table S8. Global compilation of diffusive benthic dissolved iron flux data reference list.

No.	Reference	Region
1	Ciceri et al. (1992)	Mediterranean Sea
2	Elrod et al. (2004)	North East Pacific, California margin
3	Herbert et al. (2021)	Arctic, Svalbard fjords
4	Herbert et al. (2022)	Arctic, Svalbard fjords
5	Homoky et al. (2012)	North East Pacific, California and Oregon shelves
6	Kalnejais et al. (2015)	North West Atlantic, coastal Massachusetts
7	Noffke et al. (2012)	South East Pacific, Peruvian margin
8	Plass et al. (2020)	South East Pacific, Peruvian margin
9	Schroller-Lomnitz et al. (2019)	North East Atlantic, Mauritanian margin
10	Shi et al. (2019)	North West Pacific
11	Turetta et al. (2005)	Mediterranean Sea
12	Warnken et al. (2001)	North West Atlantic, Texas bay
13	Wehrmann et al. (2014)	Arctic, Svalbard fjords
14	This study	Chilean Patagonia, Baker-Martinez Fjord Complex

Table S9. Global compilation of diffusive benthic dissolved silicon flux data reference list.

No.	Reference	Region
1	Aller et al. (1985)	North West Pacific, East China Sea
2	Berelson et al. (1987)	North East Pacific, California basins
3	Berelson et al. (1990)	Equatorial Pacific
4	Bertuzzi et al. (1996)	Mediterranean Sea
5	Cassarino et al. (2020)	Southern Ocean, West Antarctic Peninsula
6	Closset et al. (2022)	Southern Ocean, Pacific sector
7	Devol and Christensen (1993)	North East Pacific, Washington margin
8	Grandel et al. (2000)	North Indian Ocean, Arabian Sea
9	Hammond et al. (2000)	North East Pacific, California margin
10	Hensen et al. (1998)	Equatorial and South Atlantic
11	Holstein and Hensen (2010)	Equatorial and South Atlantic
12	Hou et al. (2019)	North Pacific
13	King et al. (2000)	South Atlantic
14	Koning et al. (1997)	North West Indian Ocean, Somali basin
15	Liu et al. (2005)	North West Pacific, East China Sea
16	März et al. (2015)	Arctic Ocean
17	McManus et al. (1995)	Equatorial Pacific
18	McManus et al. (2003)	North East and South East Pacific
19	Ng et al. (2020)	North Atlantic, Labrador Sea and Greenland coast
20	Pratihary et al. (2014)	North Indian Ocean, Arabian Sea
21	Rabouille et al. (1997)	South Pacific
22	Ragueneau et al. (2001)	North East Atlantic
23	Ragueneau et al. (2009)	South East Atlantic, Congo margin
24	Rutgers van der Loeff and van Bennekom (1989)	Southern Ocean, Atlantic sector
25	Sayles et al. (1996)	North West Atlantic
26	Sayles et al. (2001)	Southern Ocean, Pacific sector
27	Schlüter et al. (1998)	Southern Ocean, Atlantic sector
28	Shibamoto and Harada (2010)	North West Pacific
29	Thibodeau et al. (2010)	North West Atlantic, Laurentian Channel
30	Ward et al. (2022a)	Arctic Ocean, Barents Sea
31	Ziebis et al. (2012)	North Atlantic, Mid-Atlantic Ridge
32	This study	Chilean Patagonia, Baker-Martinez Fjord Complex

References

Aller, R. C., Mackin, J. E., Ullman, W. J., Chen-Hou, W., Shing-Min, T., Jian-Cai, J., et al. (1985). Early chemical diagenesis, sediment-water solute exchange, and storage of reactive organic matter near the mouth of the Changjiang, East China Sea. *Continental Shelf Research, 4*(1-2), 227-251. doi:https://doi.org/10.1016/0278-4343(85)90031-7

Berelson, W. M., Hammond, D. E., & Johnson, K. S. (1987). Benthic fluxes and the cycling of biogenic silica and carbon in two southern California borderland basins. *Geochimica Et Cosmochimica Acta, 51*(6), 1345-1363. doi:https://doi.org/10.1016/0016-7037(87)90320-6

Berelson, W. M., Hammond, D. E., O'Neill, D., Xu, X. M., Chin, C., & Zukin, J. (1990). Benthic fluxes and pore water studies from sediments of the central equatorial north Pacific: Nutrient diagenesis. *Geochimica Et Cosmochimica Acta, 54*(11), 3001-3012. doi:https://doi.org/10.1016/0016-7037(90)90117-4

Bertuzzi, A., Faganeli, J., & Bflmbati, A. (1996). Annual variation of benthic nutrient fluxes in shallow coastal waters (Gulf of Trieste, northern Adriatic Sea). *Marine Ecology, 17*(1-3), 261-278. doi:https://doi.org/10.1111/j.1439-0485.1996.tb00507.x

Boldt, K. V. (2014). *Fjord sedimentation during the rapid retreat of tidewater glaciers: observations and modeling (PhD thesis).* (Doctor of Philosophy). University of Washington,

Boudreau, B. P. (1996). The diffusive tortuosity of fine-grained unlithified sediments. *Geochimica Et Cosmochimica Acta, 60*(16), 3139-3142. doi:https://doi.org/10.1016/0016-7037(96)00158-5

Cassarino, L., Hendry, K. R., Henley, S. F., MacDonald, E., Arndt, S., Freitas, F. S., et al. (2020). Sedimentary nutrient supply in productive hot spots off the West Antarctic Peninsula revealed by silicon isotopes. *Global Biogeochemical Cycles, 34*(12), e2019GB006486. doi:https://doi.org/10.1029/2019GB006486

Ciceri, G., Maran, C., Martinotti, W., & Queirazza, G. (1992). Geochemical cycling of heavy metals in a marine coastal area: benthic flux determination from pore water profiles and in situ measurements using benthic chambers. *Hydrobiologia, 235*(1), 501-517. doi:https://doi.org/10.1007/BF00026238

Closset, I., Brzezinski, M. A., Cardinal, D., Dapoigny, A., Jones, J. L., & Robinson, R. S. (2022). A silicon isotopic perspective on the contribution of diagenesis to the sedimentary silicon budget in the Southern Ocean. *Geochimica Et Cosmochimica Acta, 327*, 298-313. doi:https://doi.org/10.1016/j.gca.2022.04.010

De Wilde, E. (2016). *Fjord Sedimentary Record of the Recent and Rapid Retreat of Jorge Montt Glacier, Chilean Patagonia (Masters thesis).* Ghent University,

Devol, A. H., & Christensen, J. P. (1993). Benthic fluxes and nitrogen cycling in sediments of the continental margin of the eastern North Pacific. *Journal of Marine Research, 51*(2), 345-372. doi:https://doi.org/10.1357/0022240933223765

Ding, T., Wan, D., Bai, R., Zhang, Z., Shen, Y., & Meng, R. (2005). Silicon isotope abundance ratios and atomic weights of NBS-28 and other reference materials. *Geochimica Et Cosmochimica Acta, 69*(23), 5487-5494. doi:https://doi.org/10.1016/j.gca.2005.06.015

Elrod, V. A., Berelson, W. M., Coale, K. H., & Johnson, K. S. (2004). The flux of iron from continental shelf sediments: A missing source for global budgets. *Geophysical Research Letters, 31*(12), L12307. doi:https://doi.org/10.1029/2004GL020216

Freitas, F. S., Hendry, K. R., Henley, S. F., Faust, J. C., Tessin, A. C., Stevenson, M. A., et al. (2020). Benthic-pelagic coupling in the Barents Sea: an integrated data-model framework. *Philosophical Transactions of the Royal Society A: Mathematical, Physical and Engineering Sciences, 378*(2181), 20190359. doi:https://doi.org/10.1098/rsta.2019.0359

Grandel, S., Rickert, D., Schlüter, M., & Wallmann, K. (2000). Pore-water distribution and quantification of diffusive benthic fluxes of silicic acid, nitrate and phosphate in surface sediments of the deep Arabian Sea. *Deep Sea Research Part II: Topical Studies in Oceanography, 47*(14), 2707-2734. doi:https://doi.org/10.1016/S0967-0645(00)00046-1

Hammond, D. E., McManus, J., Berelson, W. M., Meredith, C., Klinkhammer, G. P., & Coale, K. H. (2000). Diagenetic fractionation of Ge and Si in reducing sediments: the missing Ge sink and a possible mechanism to cause glacial/interglacial variations in oceanic Ge/Si. *Geochimica Et Cosmochimica Acta, 64*(14), 2453-2465. doi:https://doi.org/10.1016/S0016-7037(00)00362-8

Hatton, J. E., Hendry, K. R., Hawkings, J. R., Wadham, J. L., Kohler, T. J., Stibal, M., et al. (2019). Investigation of subglacial weathering under the Greenland Ice Sheet using silicon isotopes. *Geochimica Et Cosmochimica Acta, 247*, 191-206. doi:https://doi.org/10.1016/j.gca.2018.12.033

Hawkings, J. R., Hatton, J. E., Hendry, K. R., de Souza, G. F., Wadham, J. L., Ivanovic, R., et al. (2018). The silicon cycle impacted by past ice sheets. *Nature Communications, 9*(1), 3210. doi:https://doi.org/10.1038/s41467-018-05689-1

Hensen, C., Landenberger, H., Zabel, M., & Schulz, H. D. (1998). Quantification of diffusive benthic fluxes of nitrate, phosphate, and silicate in the southern Atlantic Ocean. *Global Biogeochemical Cycles, 12*(1), 193-210. doi:https://doi.org/10.1029/97GB02731

Herbert, L. C., Michaud, A. B., Laufer-Meiser, K., Hoppe, C. J. M., Zhu, Q., Aller, R. C., et al. (2022). Tight benthic-pelagic coupling drives seasonal and interannual changes in iron-sulfur cycling in Arctic fjord sediments (Kongsfjorden, Svalbard). *Journal of Marine Systems, 225*, 103645. doi:https://doi.org/10.1016/j.jmarsys.2021.103645

Herbert, L. C., Zhu, Q., Michaud, A. B., Laufer-Meiser, K., Jones, C. K., Riedinger, N., et al. (2021). Benthic iron flux influenced by climate-sensitive interplay between organic carbon availability and sedimentation rate in Arctic fjords. *Limnology and Oceanography, 66*(9), 3374-3392. doi:https://doi.org/10.1002/lno.11885

Holstein, J. M., & Hensen, C. (2010). Microbial mediation of benthic biogenic silica dissolution. *Geo-Marine Letters, 30*(5), 477-492. doi:https://doi.org/10.1007/s00367-009-0181-3

Homoky, W. B., Severmann, S., McManus, J., Berelson, W. M., Riedel, T. E., Statham, P. J., et al. (2012). Dissolved oxygen and suspended particles regulate the benthic flux of iron from continental margins. *Marine Chemistry, 134-135*, 59-70. doi:https://doi.org/10.1016/j.marchem.2012.03.003

Hou, Y., Hammond, D. E., Berelson, W. M., Kemnitz, N., Adkins, J. F., & Lunstrum, A. (2019). Spatial patterns of benthic silica flux in the North Pacific reflect upper ocean production. *Deep Sea Research Part I: Oceanographic Research Papers, 148*, 25-33. doi:https://doi.org/10.1016/j.dsr.2019.04.013

Kalnejais, L. H., Martin, W. R., & Bothner, M. H. (2015). Porewater dynamics of silver, lead and copper in coastal sediments and implications for benthic metal fluxes. *Science of The Total Environment, 517*, 178-194. doi:https://doi.org/10.1016/j.scitotenv.2015.02.011

Khalil, K., Rabouille, C., Gallinari, M., Soetaert, K., DeMaster, D. J., & Ragueneau, O. (2007). Constraining biogenic silica dissolution in marine sediments: A comparison between diagenetic models and experimental dissolution rates. *Marine Chemistry, 106*(1), 223-238. doi:https://doi.org/10.1016/j.marchem.2006.12.004

King, S. L., Froelich, P. N., & Jahnke, R. A. (2000). Early diagenesis of germanium in sediments of the Antarctic South Atlantic: in search of the missing Ge sink. *Geochimica Et Cosmochimica Acta, 64*(8), 1375-1390. doi:https://doi.org/10.1016/S0016-7037(99)00406-8

Koning, E., Brummer, G.-J., Van Raaphorst, W., Van Bennekom, J., Helder, W., & Van Iperen, J. (1997). Settling, dissolution and burial of biogenic silica in the sediments off Somalia (northwestern Indian Ocean). *Deep Sea Research Part II: Topical Studies in Oceanography, 44*(6-7), 1341-1360. doi:https://doi.org/10.1016/S0967-0645(97)00018-0

Laufer-Meiser, K., Michaud, A. B., Maisch, M., Byrne, J. M., Kappler, A., Patterson, M. O., et al. (2021). Potentially bioavailable iron produced through benthic cycling in glaciated Arctic fjords of Svalbard. *Nature Communications, 12*(1), 1349. doi:https://doi.org/10.1038/s41467-021-21558-w

Liu, S., Zhang, J., & Li, R. (2005). Ecological significance of biogenic silica in the East China Sea. *Marine Ecology Progress Series, 290*, 15-26. doi:https://doi.org/10.3354/meps290015

Loucaides, S., Van Cappellen, P., Roubeix, V., Moriceau, B., & Ragueneau, O. (2012). Controls on the Recycling and Preservation of Biogenic Silica from Biomineralization to Burial. *Silicon, 4*(1), 7-22. doi:https://doi.org/10.1007/s12633-011-9092-9

März, C., Meinhardt, A. K., Schnetger, B., & Brumsack, H. J. (2015). Silica diagenesis and benthic fluxes in the Arctic Ocean. *Marine Chemistry, 171*, 1-9. doi:https://doi.org/10.1016/j.marchem.2015.02.003

McManus, J., Hammond, D. E., Berelson, W. M., Kilgore, T. E., Demaster, D. J., Ragueneau, O. G., et al. (1995). Early diagenesis of biogenic opal: Dissolution rates, kinetics, and paleoceanographic implications. *Deep Sea Research Part II: Topical Studies in Oceanography, 42*(2-3), 871-903. doi:https://doi.org/10.1016/0967-0645(95)00035-O

McManus, J., Hammond, D. E., Cummins, K., Klinkhammer, G. P., & Berelson, W. M. (2003). Diagenetic Ge-Si fractionation in continental margin environments: further evidence for a nonopal Ge sink. *Geochimica Et Cosmochimica Acta, 67*(23), 4545-4557. doi:https://doi.org/10.1016/S0016-7037(03)00385-5

Millero, F. J., Sotolongo, S., & Izaguirre, M. (1987). The oxidation kinetics of Fe(II) in seawater. *Geochimica Et Cosmochimica Acta, 51*(4), 793-801. doi:https://doi.org/10.1016/0016-7037(87)90093-7

Ng, H. C., Cassarino, L., Pickering, R. A., Woodward, E. M. S., Hammond, S. J., & Hendry, K. R. (2020). Sediment efflux of silicon on the Greenland margin and implications for the marine silicon cycle. *Earth and Planetary Science Letters, 529*, 115877. doi:https://doi.org/10.1016/j.epsl.2019.115877

Noffke, A., Hensen, C., Sommer, S., Scholz, F., Bohlen, L., Mosch, T., et al. (2012). Benthic iron and phosphorus fluxes across the Peruvian oxygen minimum zone. *Limnology and Oceanography, 57*(3), 851-867. doi:https://doi.org/10.4319/lo.2012.57.3.0851

Oleszczuk, B., Michaud, E., Morata, N., Renaud, P. E., & Kędra, M. (2019). Benthic macrofaunal bioturbation activities from shelf to deep basin in spring to summer transition in the Arctic Ocean. *Marine Environmental Research, 150*, 104746. doi:https://doi.org/10.1016/j.marenvres.2019.06.008

Opfergelt, S., & Delmelle, P. (2012). Silicon isotopes and continental weathering processes: Assessing controls on Si transfer to the ocean. *Comptes Rendus Geoscience, 344*(11), 723-738. doi:https://doi.org/10.1016/j.crte.2012.09.006

Piret, L., Bertrand, S., Hawkings, J., Kylander, M. E., Torrejón, F., Amann, B., et al. (2021). High-resolution fjord sediment record of a receding glacier with growing intermediate proglacial lake (Steffen Fjord, Chilean Patagonia). *Earth Surface Processes and Landforms, 46*, 239-251. doi:https://doi.org/10.1002/esp.5015

Piret, L., Bertrand, S., Nguyen, N., Hawkings, J., Rodrigo, C., & Wadham, J. (2022). Long-lasting impacts of a 20th century glacial lake outburst flood on a Patagonian fjord-river system (Pascua River). *Geomorphology, 399*, 108080. doi:https://doi.org/10.1016/j.geomorph.2021.108080

Plass, A., Schlosser, C., Sommer, S., Dale, A. W., Achterberg, E. P., & Scholz, F. (2020). The control of hydrogen sulfide on benthic iron and cadmium fluxes in the oxygen minimum zone off Peru. *Biogeosciences, 17*(13), 3685-3704. doi:https://doi.org/10.5194/bg-17-3685-2020

Pratihary, A., Naqvi, S., Narvenkar, G., Kurian, S., Naik, H., Naik, R., et al. (2014). Benthic mineralization and nutrient exchange over the inner continental shelf of western India. *Biogeosciences, 11*, 2771-2791. doi:https://doi.org/10.5194/bg-11-2771-2014

Pryer, H. V., Hawkings, J. R., Wadham, J. L., Robinson, L. F., Hendry, K. R., Hatton, J. E., et al. (2020). The Influence of Glacial Cover on Riverine Silicon and Iron Exports in Chilean Patagonia. *Global Biogeochemical Cycles, 34*(12), e2020GB006611. doi:https://doi.org/10.1029/2020GB006611

Rabouille, C., Gaillard, J.-F., Tréguer, P., & Vincendeau, M.-A. (1997). Biogenic silica recycling in surficial sediments across the Polar Front of the Southern Ocean (Indian Sector). *Deep Sea Research Part II: Topical Studies in Oceanography, 44*(5), 1151-1176. doi:https://doi.org/10.1016/S0967-0645(96)00108-7

Ragueneau, O., Gallinari, M., Corrin, L., Grandel, S., Hall, P., Hauvespre, A., et al. (2001). The benthic silica cycle in the Northeast Atlantic: annual mass balance, seasonality, and importance of non-steady-state processes for the

early diagenesis of biogenic opal in deep-sea sediments. *Progress in Oceanography, 50*(1), 171-200. doi:https://doi.org/10.1016/S0079-6611(01)00053-2

Ragueneau, O., Regaudie-de-Gioux, A., Moriceau, B., Gallinari, M., Vangriesheim, A., Baurand, F., et al. (2009). A benthic Si mass balance on the Congo margin: Origin of the 4000 m DSi anomaly and implications for the transfer of Si from land to ocean. *Deep Sea Research Part II: Topical Studies in Oceanography, 56*(23), 2197-2207. doi:https://doi.org/10.1016/j.dsr2.2009.04.003

Rebreanu, L., Vanderborght, J.-P., & Chou, L. (2008). The diffusion coefficient of dissolved silica revisited. *Marine Chemistry, 112*(3), 230-233. doi:https://doi.org/10.1016/j.marchem.2008.08.004

Rickert, D. (2000). Dissolution kinetics of biogenic silica in marine environments. *Berichte zur Polarforschung (Reports on Polar Research), 351*.

Rutgers van der Loeff, M. M., & van Bennekom, A. J. (1989). Weddell sea contributes little to silicate enrichment in Antarctic Bottom Water. *Deep Sea Research Part A. Oceanographic Research Papers, 36*(9), 1341-1357. doi:http://dx.doi.org/10.1016/0198-0149(89)90087-3

Sayles, F. L., Deuser, W. G., Goudreau, J. E., Dickinson, W. H., Jickells, T. D., & King, P. (1996). The benthic cycle of biogenic opal at the Bermuda Atlantic Time Series site. *Deep Sea Research Part I: Oceanographic Research Papers, 43*(4), 383-409. doi:https://doi.org/10.1016/0967-0637(96)00027-1

Sayles, F. L., Martin, W. R., Chase, Z., & Anderson, R. F. (2001). Benthic remineralization and burial of biogenic SiO2, CaCO3, organic carbon, and detrital material in the Southern Ocean along a transect at 170 West. *Deep Sea Research Part II: Topical Studies in Oceanography, 48*(19-20), 4323-4383. doi:https://doi.org/10.1016/S0967-0645(01)00091-1

Schlüter, M., Rutgers van der Loeff, M. M., Holby, O., & Kuhn, G. (1998). Silica cycle in surface sediments of the South Atlantic. *Deep Sea Research Part I: Oceanographic Research Papers, 45*(7), 1085-1109. doi:https://doi.org/10.1016/S0967-0637(98)00006-5

Schroller-Lomnitz, U., Hensen, C., Dale, A. W., Scholz, F., Clemens, D., Sommer, S., et al. (2019). Dissolved benthic phosphate, iron and carbon fluxes in the Mauritanian upwelling system and implications for ongoing deoxygenation. *Deep Sea Research Part I: Oceanographic Research Papers, 143*, 70-84. doi:https://doi.org/10.1016/j.dsr.2018.11.008

Schulz, H. D., & Zabel, M. (2006). *Marine geochemistry* (Vol. 2): Springer.

Shi, X., Wei, L., Hong, Q., Liu, L., Wang, Y., Shi, X., et al. (2019). Large benthic fluxes of dissolved iron in China coastal seas revealed by 224Ra/228Th disequilibria. *Geochimica Et Cosmochimica Acta, 260*, 49-61. doi:https://doi.org/10.1016/j.gca.2019.06.026

Shibamoto, Y., & Harada, K. (2010). Silicon flux and distribution of biogenic silica in deep-sea sediments in the western North Pacific Ocean. *Deep Sea Research Part I: Oceanographic Research Papers, 57*(2), 163-174. doi:https://doi.org/10.1016/j.dsr.2009.10.009

Thibodeau, B., Lehmann, M. F., Kowarzyk, J., Mucci, A., Gélinas, Y., Gilbert, D., et al. (2010). Benthic nutrient fluxes along the Laurentian Channel: impacts on the N budget of the St. Lawrence marine system. *Estuarine, Coastal and Shelf Science, 90*(4), 195-205. doi:https://doi.org/10.1016/j.ecss.2010.08.015

Turetta, C., Capodaglio, G., Cairns, W., Rabar, S., & Cescon, P. (2005). Benthic fluxes of trace metals in the lagoon of Venice. *Microchemical Journal, 79*(1-2), 149-158. doi:https://doi.org/10.1016/j.microc.2004.06.003

Vandekerkhove, E., Bertrand, S., Torrejón, F., Kylander, M. E., Reid, B., & Saunders, K. M. (2021). Signature of modern glacial lake outburst floods in fjord sediments (Baker River, southern Chile). *Sedimentology*, doi: 10.1111/sed.12874. doi:https://doi.org/10.1111/sed.12874

Ward, J. P. J., Hendry, K. R., Arndt, S., Faust, J. C., Freitas, F. S., Henley, S. F., et al. (2022a). Stable Silicon Isotopes Uncover a Mineralogical Control on the Benthic Silicon Cycle in the Arctic Barents Sea. *Geochimica Et Cosmochimica Acta, 329*, 206-230. doi:https://doi.org/10.1016/j.gca.2022.05.005

Ward, J. P. J., Hendry, K. R., Arndt, S., Faust, J. C., Freitas, F. S., Henley, S. F., et al. (2022b). Benthic Silicon Cycling in the Arctic Barents Sea: a Reaction-Transport Model Study. *Biogeosciences*, *19*, 3445–3467. doi:https://doi.org/10.5194/bg-19-3445-2022

Warnken, K. W., Gill, G. A., Griffin, L. L., & Santschi, P. H. (2001). Sediment-water exchange of Mn, Fe, Ni and Zn in Galveston Bay, Texas. *Marine Chemistry, 73*(3), 215-231. doi:https://doi.org/10.1016/S0304-4203(00)00108-0

Wehrmann, L. M., Formolo, M. J., Owens, J. D., Raiswell, R., Ferdelman, T. G., Riedinger, N., et al. (2014). Iron and manganese speciation and cycling in glacially influenced high-latitude fjord sediments (West Spitsbergen, Svalbard): Evidence for a benthic recycling-transport mechanism. *Geochimica Et Cosmochimica Acta, 141*, 628-655. doi:https://doi.org/10.1016/j.gca.2014.06.007

Wetzel, F., de Souza, G. F., & Reynolds, B. C. (2014). What controls silicon isotope fractionation during dissolution of diatom opal? *Geochimica Et Cosmochimica Acta, 131*, 128-137. doi:https://doi.org/10.1016/j.gca.2014.01.028

Willey, J. D. (1974). The effect of pressure on the solubility of amorphous silica in seawater at 0°C. *Marine Chemistry, 2*(4), 239-250. doi:https://doi.org/10.1016/0304-4203(74)90018-8

Ziebis, W., McManus, J., Ferdelman, T., Schmidt-Schierhorn, F., Bach, W., Muratli, J., et al. (2012). Interstitial fluid chemistry of sediments underlying the North Atlantic gyre and the influence of subsurface fluid flow. *Earth and Planetary Science Letters, 323*, 79-91. doi:https://doi.org/10.1016/j.epsl.2012.01.018
